# A conserved regulatory architecture stabilizes cellular senescence across distinct triggers in human fibroblasts

**DOI:** 10.1007/s11357-026-02297-6

**Published:** 2026-05-07

**Authors:** Mohd Shahzaib, Domenico Aprile, Tiziana Squillaro, Nicola Alessio, Gianfranco Peluso, Giovanni Di Bernardo, Umberto Galderisi

**Affiliations:** 1 Department of Experimental Medicine, Biotechnology and Molecular Biology Section, Luigi Vanvitelli Campania University, 80138 Naples, Italy; 2 Department of Life Sciences, Health and Health Professions, Link Campus University, 00165 Rome, Italy; 3 Faculty of Medicine and Surgery, Saint Camillus International, University of Health Sciences, Rome, Italy; 4 Sbarro Health Research Organization, Temple University, Philadelphia, PA 19122 USA; 5 Genome and Stem Cell Center (GENKÖK), Erciyes University, Kayseri, Turkey

**Keywords:** Cellular senescence, Human fibroblasts, Transcriptomics, Gene Ontology, Reactome, Network biology

## Abstract

Cellular senescence arises through replicative exhaustion or acute stress, yet whether these distinct triggers share a reproducible transcriptional organization has remained unresolved. Seven public human fibroblast RNA-seq datasets were integrated across both trigger types, moving from differential expression through Gene Ontology and Reactome enrichment to protein–protein interaction network embedding within a single harmonized framework. Both triggers converged on concordant repression of replication and chromatin programs alongside induction of inflammatory and extracellular matrix outputs. Cross-trigger combination identified 263 commonly repressed and 112 commonly induced GO terms, with consistently higher enrichment scores in the repressed set, and 145 versus 13 shared Reactome pathways. Conserved induction of calcium ion homeostasis and membrane potential regulation, and conserved repression of RHO GTPase-Formin signaling, extended the shared program beyond canonical SASP biology into dimensions not previously described in human fibroblasts. Interactome embedding, applied here for the first time in a cross-trigger senescence framework, identified ten genes occupying both induced and repressed neighborhoods simultaneously, a structural layer invisible to enrichment analysis alone. ELAVL1 coordinates SASP output and growth arrest post-transcriptionally, while suppressed PARP1 alongside active ATM defines a self-reinforcing damage configuration. These results provide a quantitative cross-study reference and a structured basis for senotherapeutic candidate prioritization.

**Graphical abstract:**

Distinct senescence triggers, including replicative exhaustion and acute stress, converge on a shared senescent state in human fibroblasts. This state is defined by a conserved regulatory architecture comprising interconnected modules of checkpoint enforcement, inflammatory/SASP signaling, and repression of replication and chromatin programs. A small set of bridging nodes links these modules, supporting a stable and self-maintaining network organization of cellular senescence.

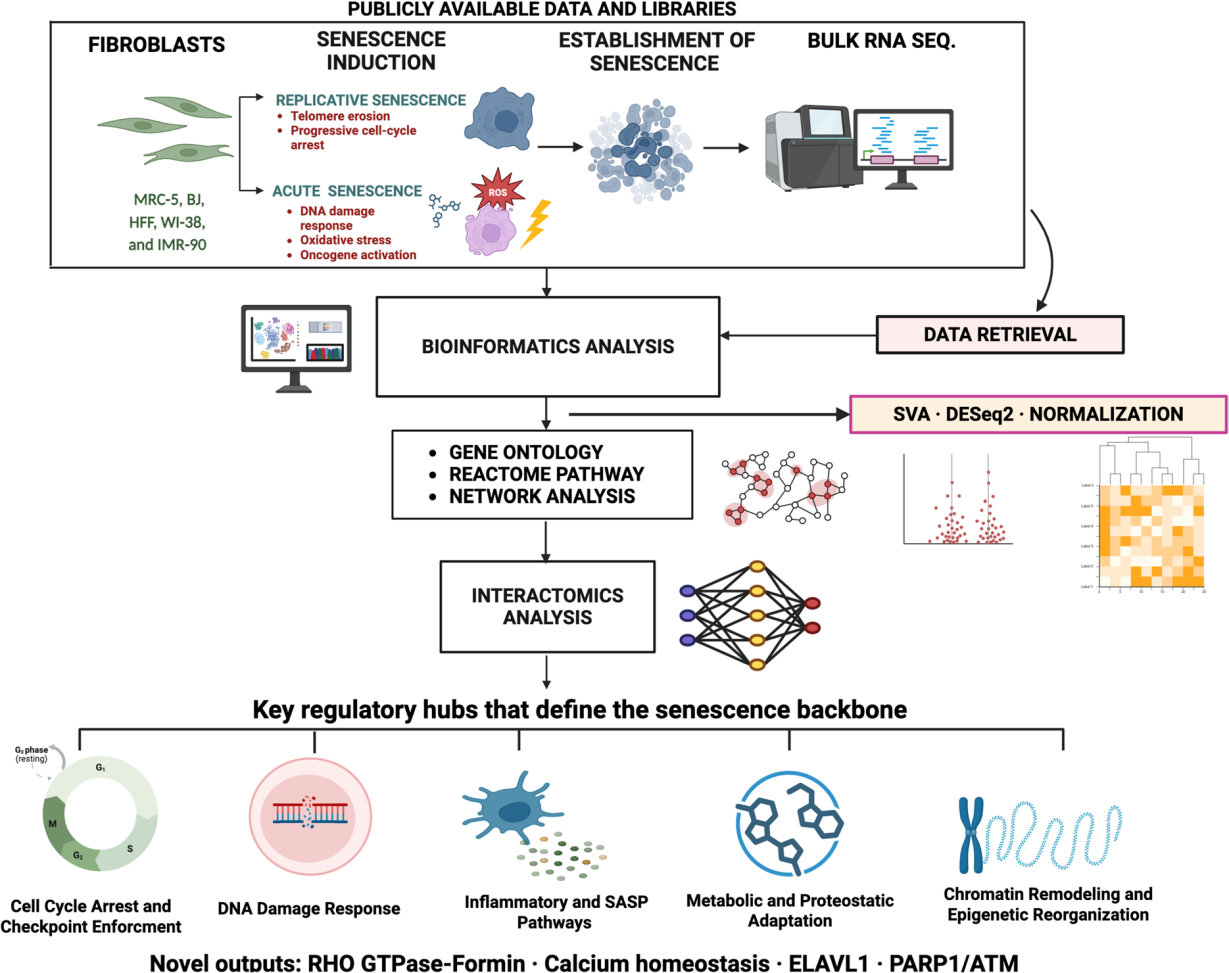

**Supplementary Information:**

The online version contains supplementary material available at 10.1007/s11357-026-02297-6.

## Introduction

Cellular senescence is a regulated stress response triggered by diverse stimuli, including telomere erosion, persistent DNA damage, chromatin alterations, and oncogenic signaling. While senescence plays essential physiological roles in tissue repair, embryonic development, and tumor suppression, its chronic accumulation contributes to organismal aging, inflammaging, and the progression of age-associated diseases such as fibrosis, neurodegeneration, and cancer [1].

Human fibroblasts represent one of the most extensively characterized and experimentally accessible models of cellular senescence. These cells undergo robust and reproducible senescence programs in response to both replicative exhaustion and acute stress, making them a central system for studying the molecular determinants of senescence and its impact on tissue homeostasis. Recent studies further emphasize that fibroblast senescence is not only a hallmark of tissue aging but also a key driver of extracellular matrix remodeling, chronic inflammation, and microenvironmental dysfunction across multiple physiological and pathological contexts [2].

Although hundreds of transcriptomic studies have characterized senescent cells in various models [3], there is a lack of a common rigorously cross-validated framework capable of integrating replicative senescence and stress or damage-induced (acute) senescence [4].

A major challenge in senescence research is the absence of a universal marker. Instead, senescence is defined by a combination of molecular and functional features, including stable cell cycle arrest, activation of DNA damage responses, chromatin reorganization, and the acquisition of a senescence-associated secretory phenotype (SASP) [5].

Transcriptomic profiling has been instrumental in identifying recurrent features of senescence; however, these studies also highlight substantial variability depending on cell type, inducing stimulus, and experimental context [6]. As a result, individual studies often capture only partial aspects of the senescence program [7].

Meta-analytical approaches provide a powerful strategy to overcome these limitations by integrating independent datasets and identifying reproducible features across experimental systems [8]. Previous efforts have primarily focused on differential expression and functional enrichment analyses, revealing common trends such as the upregulation of inflammatory and extracellular matrix pathways and the repression of DNA replication and repair processes [9, 10]. While informative, these approaches remain largely descriptive and do not fully address how these molecular changes are organized into a stable cellular state [11].

A central unresolved question in cell biology is how senescence is stabilized and maintained despite being initiated by distinct upstream triggers such as replicative exhaustion and acute stress. Although these conditions activate partially divergent signaling cascades, senescent cells consistently converge on a shared phenotype characterized by durable growth arrest, chromatin remodeling, and sustained inflammatory signaling [9]. We hypothesized that this convergence reflects the emergence of a conserved regulatory architecture that constrains cellular behavior into a stable and self-maintaining state.

Here, we define conserved regulatory architecture as the reproducible organization of senescence into interconnected functional modules, encompassing checkpoint enforcement, inflammatory and extracellular matrix activation, and repression of replication and chromatin programs, together with a limited set of high-centrality regulatory nodes that bridge these modules across conditions.

To identify this architecture, we performed an integrative meta-analysis of transcriptomic datasets derived from replicative and stress-induced senescence in human fibroblasts. By combining differential expression analysis with functional enrichment, pathway reconstruction, and interactomic modeling, we aimed to isolate invariant network components that persist across senescence modalities [12]. This systems-level approach enables the identification of a shared regulatory backbone underlying senescence maintenance [13].

Understanding the conserved architecture of cellular senescence is critical for the rational design of interventions targeting senescent cells. By identifying network components that are consistently preserved across conditions, this work provides a framework for prioritizing candidate targets for future functional validation and therapeutic modulation in aging and age-related diseases.

## Methods

### Inclusion criteria for study selection

The studies were considered according to the following criteria to maintain methodological consistency and biological relevance.

Peer reviewed. Only peer-reviewed studies were eligible, ensuring scientific rigor and strict research standards.

Mammalian model systems. We deemed experiments in mammalian cells an area to focus on for the human relevance of cellular aging mechanisms and enhanced translational value.

Transcriptome-wide profiling. Studies need to utilize bulk RNA sequencing (RNA-seq) and measure global expression levels to capture a genome-wide perspective of transcriptional alterations. The raw or processed RNA-seq data used in the work should have been publicly available or made accessible by the authors for reproducibility.

Senescence-specific orientation. Our literature survey was focused on primary research works that specifically addressed cellular senescence resulting from replicative exhaustion (e.g., extended passaging) or acute stressors (e.g., oxidative, genotoxic, and oncogenic insults).

Transparency document. The datasets, fully annotated with metadata, including experimental conditions used, protocol to induce senescence, and number of biological replicates, were considered in support of cross-study comparisons.

### Search for query strategy

We conducted a search to identify relevant bulk RNA sequencing studies and data from PubMed. The following keyword was applied across all databases: ((cellular senescence [Title/Abstract]) OR (cellular aging [Title/Abstract]) OR (senescence [Title/Abstract])) AND (transcriptome analysis [Title/Abstract]). Additionally, we executed a query to locate senescence-related transcriptomic data specifically within the Gene Expression Omnibus (GEO) and ArrayExpress databases. This query targeted records with titles containing “senescence” and “Homo sapiens,” focusing on articles published in English.

### Data collection

We conducted a meticulous review of the search results to select articles that met our inclusion criteria and evaluated their suitability for this study. Bulk RNA sequencing data were obtained from the chosen studies. For quantitative analysis, we included RNA-seq data identified exclusively in senescent fibroblast cells, which showed significant enrichment. The gene annotation file was downloaded from the NCBI database to align the Gene ID with its corresponding gene symbol and name. Additionally, we compiled experimental data such as species, cell type, stress type, and duration since stress induction (Sup Fig. 1, Sup Fig. 2).

### Data curation and selection process from PubMed and general RNA-seq databases

A total of 344 research papers were identified from PubMed, with a focus on model organisms and humans. Human research accounts for 26.2%, focusing on biology, diseases, and therapies. Mouse studies make up 15.4%, serving as key models in genetics and biomedicine. The majority, 58.4%, involve other species, emphasizing research in agriculture, ecology, and evolution (Sup Fig. 1 A). We scrupulously reviewed these studies to select those that met our inclusion criteria. Human studies dominate, with 90 papers utilizing RNA sequencing: 48 on bulk RNA-seq, 30 on microarrays, 6 on single-cell RNA sequencing (scRNA-Seq), and 2 combining bulk and scRNA-Seq (Sup. Fig. 1B). Publicly available data includes 15 bulk RNA-seq studies and 2 combined datasets, hosted in 13 databases for bulk RNA-seq and 2 for combined analyses. The selected bulk RNA sequencing data were exclusively from senescent fibroblast cells, showing significant enrichment. All data related to number of papers and species type are present in Supplementary Figure 1.

For further analysis from PubMed papers, we selected 48 bulk RNA sequencing datasets categorized by human cell types, along with 2 datasets that combine scRNA-Seq and bulk RNA-seq. The identified cell types include mesenchymal stromal cells, fibroblasts, cancer cells, stem cells, and other cells (immune cells, endometrial cells, pancreatic cells, kidney cortex cells, etc.), with detailed numbers and types provided in Supplementary Figure 1C.

After thorough consideration, we chose two published bulk RNA-seq datasets GSE63577 and GSE64553 in NCBI GEO for analysis, downloaded the data, and started data pre-processing. These are fibroblast cell strain that were linked to replicative senescence: BJ, MRC5, WI38, and human foreskin. Particularly, GSE63577 is composed of BJ fibroblasts, MRC5, and WI38 cell lines as well as human foreskin fibroblast cell strain; GSE64553 contains human foreskin fibroblast cell strain. They selected both datasets on the population doubling time of the cells and are thus well suited to study replicative senescence. The datasets provide insights into cellular aging across different fibroblast types, with samples divided into early and late passage conditions.

The useful datasets that could be retrieved by PubMed analysis referred mainly to senescence of human fibroblasts. We then focused our attention on this cell type.

To identify human fibroblast transcriptomic profiles capturing replicative or acute senescence, we conducted a search across public repositories. While ArrayExpress (https://www.ebi.ac.uk/biostudies/arrayexpress) yielded no datasets meeting our criteria, a comprehensive search of the GEO (https://www.ncbi.nlm.nih.gov/geo/) identified 33 candidate datasets broadly related to cellular senescence. These datasets were categorized by platform: 18 employed microarray sequencing, 14 utilized bulk RNA sequencing (RNA-seq), while 1 was for methylation profiling by array (Sup. Fig. 1D).

The selected bulk RNA-seq datasets, following the procedure above described, were prioritized according to their higher resolution, dynamic range, and suitability for cross-study comparisons. Of the 14 included RNA-seq studies, only 5 satisfied our strict inclusion criteria:

1. Human fibroblast focus: Non-fibroblast or non-human models were not included.
2. Design specific to senescence: It was necessary that there be documented antecedents of the passage to senescence (either a replicative exhaustion with high levels of population doubling or through an acute incubation with stressors: oxidative/genotoxic agents).

- 3. Data availability: Selection of studies consisting of raw or normalized RNA-seq counts, metadata treatment information, and biological replicates are included.

From this selective approach, we included datasets GSE262856, GSE222400, GSE235768, GSE130727, and GSE73458 aligned to our aim of identifying conserved transcriptional signatures of senescence while minimizing platform-specific bias (Sup. Fig. 2).

### Transcriptomic datasets and senescence models

We analyzed publicly available bulk RNA-seq datasets of human fibroblasts undergoing replicative or stress-induced senescence. Seven GEO series were included: GSE63577, GSE64553, GSE262856, GSE222400, GSE235768, GSE130727, and GSE73458. These datasets cover the fibroblast lines MRC-5, BJ, HFF, WI-38, and IMR-90.

Samples were classified into three biological conditions: (i) proliferating controls at low population-doubling levels (PDLs), (ii) replicative senescence at high PDLs with loss of mitotic capacity, and (iii) acute senescence induced by defined stressors. Replicative senescence was defined by extended passaging to high PDLs (e.g., BJPD72, WI-38PD53–55, and MRC-5PD72). Acute senescence was induced by clinically and biologically relevant insults including doxorubicin, bortezomib, oxidative stress (H_2_O_2_), and ionizing radiation, with samples collected after recovery periods allowing establishment of the senescent phenotype.

All datasets included biological replicates (duplicates or triplicates) and detailed metadata describing experimental conditions, treatments, and PDLs. Time-course data were available for selected datasets (e.g., WI-38 doxorubicin Day 0–16), enabling assessment of both senescence induction and maintenance phases.

### Data preprocessing, quality control, and normalization

Raw RNA-seq count matrices and metadata were matched through sample identifiers, and quality checks were performed to verify that the samples agreed with the experimental conditions. Lowly expressed genes were removed from the dataset with DESeq2 (version 1.48.1) [14], keeping only those features which had counts ≥ 10 in at least two samples. Differences in library sizes were adjusted by DESeq2 median-of-ratios size-factor normalization, and then, variance-stabilizing transformation (VST) was applied [15]. Batch effects were mitigated using Surrogate Variable Analysis (SVA, version 3.56.0) [16], with residual technical variation visually assessed using removeBatchEffect from limma (version 3.64.3) [17] and principal component analysis (PCA) conducted pre- and post-correction (via ggplot2, version 4.0.0) to visualize reduction in technical variability. Core Bioconductor infrastructure relied on SummarizedExperiment (1.38.1), Biobase (2.68.0), IRanges (2.42.0), S4Vectors (0.46.0), and GenomeInfoDb (1.44.2). To ensure the reproducibility of the analysis, the complete computational environment was recorded by using built-in R command *sessionInfo()* after loading all packages in Table 9 in Supplementary files. Consistent with the PCA visualization, quantitative metrics demonstrated increased condition separability and reduced batch dependence after correction (Supplementary Tables 10, 11, 12).

### Differential gene expression analysis

Differential expressions were tested in DESeq2 with the design ~ Batch + Condition, comparing acute senescence versus control and replicative senescence versus control. Log fold changes were shrunk with lfcShrink using apeglm, while retaining the original Wald *p* and false discovery rate (FDR) values. Differentially expressed genes were called at an FDR < 0.05, absolute log2 fold change of ≥ 1, and baseMean ≥ 10. Volcano plots were drawn by ggplot2 (version 4.0.0) and ggrepel (version 0.9.6); hierarchical clustering analysis was performed to visualize the top 50 differential expression genes (DEGs) in pheatmap (version 1.0.13).

### Volcano plot, Venn diagram, and heatmap

The integration of volcano plots (ggplot2 4.0.0 + ggrepel 0.9.6), Venn diagrams (VennDiagram, version 1.7.3, and ggvenn, version 0.1.10), and heatmaps (pheatmap, version 1.0.13) provides a comprehensive visualization of the transcriptional landscape in acute and replicative senescence. These tools depict the distribution and significance of differentially expressed genes, aiding in the identification of key molecular players and in providing insights into co-regulated transcriptional programs.

### Functional enrichment and pathway analysis

Gene Ontology (GO) and Reactome pathway enrichments were carried out using clusterProfiler (version 4.16.0) [18] and ReactomePA (version 1.52.0) [19], respectively. Annotation data were fetched from org.Hs.eg.db (version 3.21.0). Enriched terms (FDR < 0.05) were visualized through bar plots and comparative Venn diagrams to distinguish shared and unique pathways across senescence subtypes. For functional enrichment, we identified Gene Ontology Biological Process (GO:BP) and Reactome pathways that were significantly enriched in both acute and replicative senescence. For terms found in both models, FDR-adjusted *p* values from each were combined using Fisher’s method to produce a single meta-analytic *p* value for overlap significance. Fold enrichment is reported separately for each condition [20]. No weighted mean of log2 fold change was calculated at the pathway level. The resulting combined statistic is referred to as Fisher combined *p* throughout “Results.”

### Protein–protein interaction (PPI) network analysis

PPI networks were constructed using NETWORKANALYST (https://www.networkanalyst.ca), utilizing the Minimum IMEx Interactome Network option [21], the first-order option, and confidence score cutoff of 900 to simplify the networks to include only primary proteins (seed proteins) and their direct connections [22]. We focused on proteins with a connection degree greater than 20 and betweenness centrality over 200, highlighting the most influential players in the network.

### Statistical analysis

Statistical significance was defined as FDR < 0.05 after Benjamini–Hochberg correction. Differential expression was performed with DESeq2 (v1.48.1) in R (v4.4.2) using its negative binomial model with size-factor normalization, dispersion estimation, and Wald tests for model coefficients. Unless stated otherwise, reported *p* values are Benjamini–Hochberg adjusted. For pathway overlap analyses, *p* values were combined using Fisher’s method. Batch-correction performance was quantified in PCA space using mean silhouette width for biological condition, within- and between-condition distances, and k-nearest neighbor batch-mixing scores (fraction of same-batch neighbors), computed before and after surrogate variable adjustment.

## Results

### Replicative and stress-induced senescence converge on a shared transcriptional state

To determine whether replicative and stress-induced senescence converge on a common regulatory state, we integrated transcriptomes from human fibroblasts (MRC-5, BJ, HFF, WI-38, and IMR-90) that had entered senescence through population-doubling exhaustion or acute genotoxic, oxidative, and proteotoxic stress. After removal of low-confidence genes and correction of hidden technical variation, samples were segregated by biological state rather than by study of origin, indicating that the integrated dataset captured genuine senescence-associated transcriptional programs. This framework enabled direct comparison of replicative and acute senescence across independent experiments and provided a basis for identifying invariant and modality-specific components of the senescent transcriptome.

### Integration of independent transcriptomes reveals conserved senescence programs

**Fig. 1:**
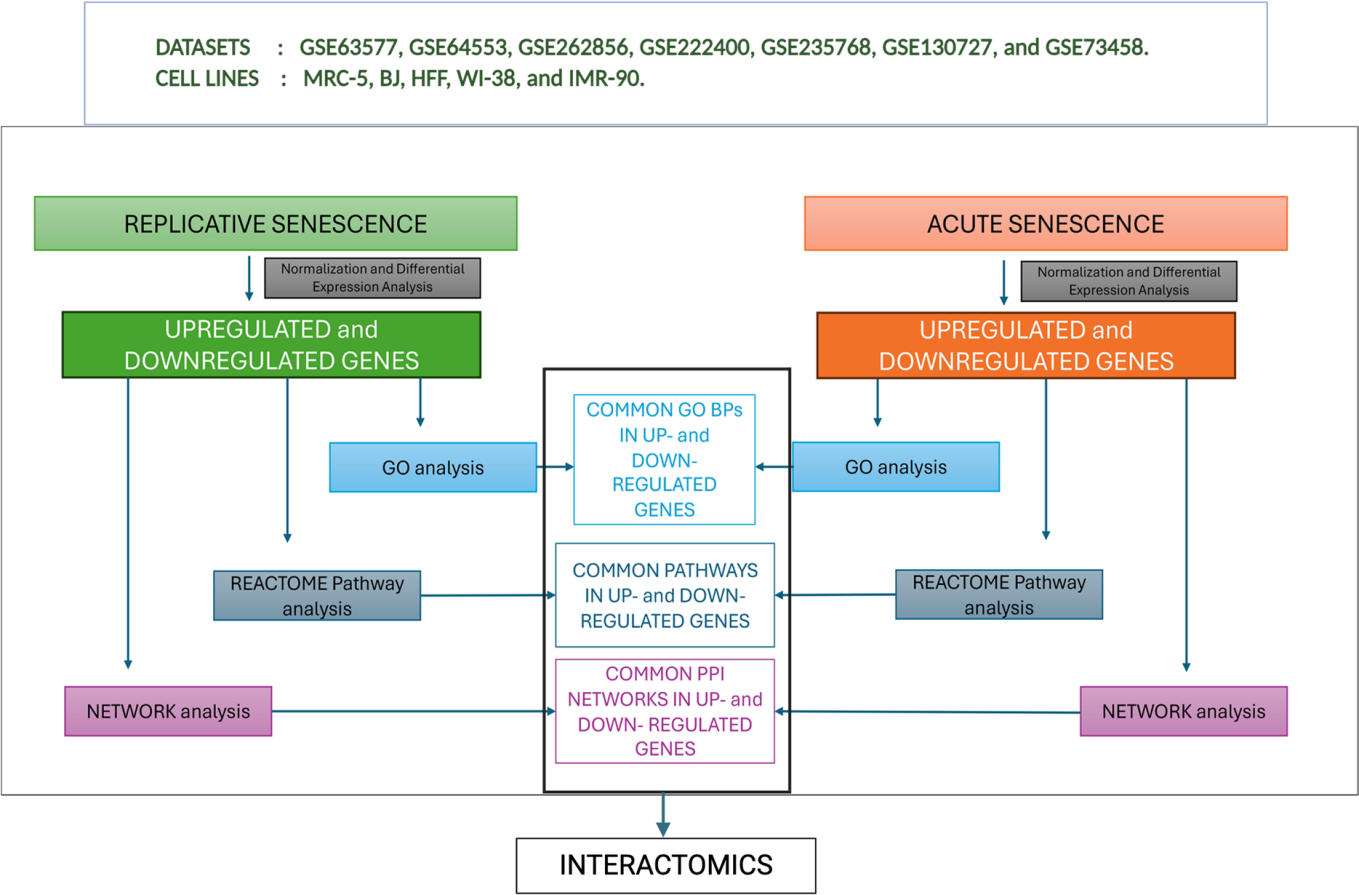
Experimental plan of the study. The diagram outlines the experimental workflow employed in the study to analyze and compare the transcriptomic profiles of replicative and acute senescence. The plan includes initial normalization and differential expression analysis for each senescence type, followed by Gene Ontology (GO) analysis, Reactome pathway analysis, and network analysis. It culminates in the integration of these analyses to identify common biological processes (BPs), pathways, and protein–protein interaction (PPI) networks that are upregulated or downregulated across both types of senescence

The meta-analysis strategies used in this study are depicted in Fig. 1. The analysis commenced with the preprocessing of gene expression data, where batch effects were corrected using SVA (version 3.56.0) to ensure cross-study comparability and minimize technical variation [16]. First, gene expression datasets from the “Methods” section detailing the senescent cells were divided into two main groups: replicative and acute senescence. Differential gene expressions were calculated using DESeq2 (version 1.48.1) [14], which allowed the recognition of genes that are upregulated or downregulated in a senescence condition consistently. Combining the DESeq2 results based on different datasets has yielded one biologically coherent and unified transcriptomic landscape, which helped to discover the conserved senescence-associated pathways even when there is heterogeneity in the experimental designs [23].

Within the harmonized expression space, a shared cross-trigger component was defined as genes concordantly dysregulated in both replicative and acute senescence. These shared gene sets were subsequently evaluated by GO and Reactome enrichment and embedded in PPI structure to identify recurrent modules and candidate interface nodes. The layered analytical strategy used here, moving from differential expression through functional enrichment to network embedding, was designed to extract information that no single layer can provide alone. GO enrichment identifies which biological processes are recurrently altered, but it does not distinguish how concentrated or dispersed those alterations are across trigger types [24].

Reactome narrows this to defined molecular pathways and reveals whether the same signaling routes are engaged regardless of how senescence was induced [25, 26].

PPI network embedding then converts gene lists into a structural map, exposing how induced and repressed programs are physically connected through shared interactors, something enrichment analysis cannot show [27]. The network revealed hub genes and suggested regulatory routes that may drive the phenotype [28]. Together, these steps provided a coherent view of transcriptomic change in senescent fibroblasts and yielded insights into the molecular basis of cellular aging.

### Correction of technical variation unmasks a conserved senescence-associated transcriptional structure

**Fig. 2:**
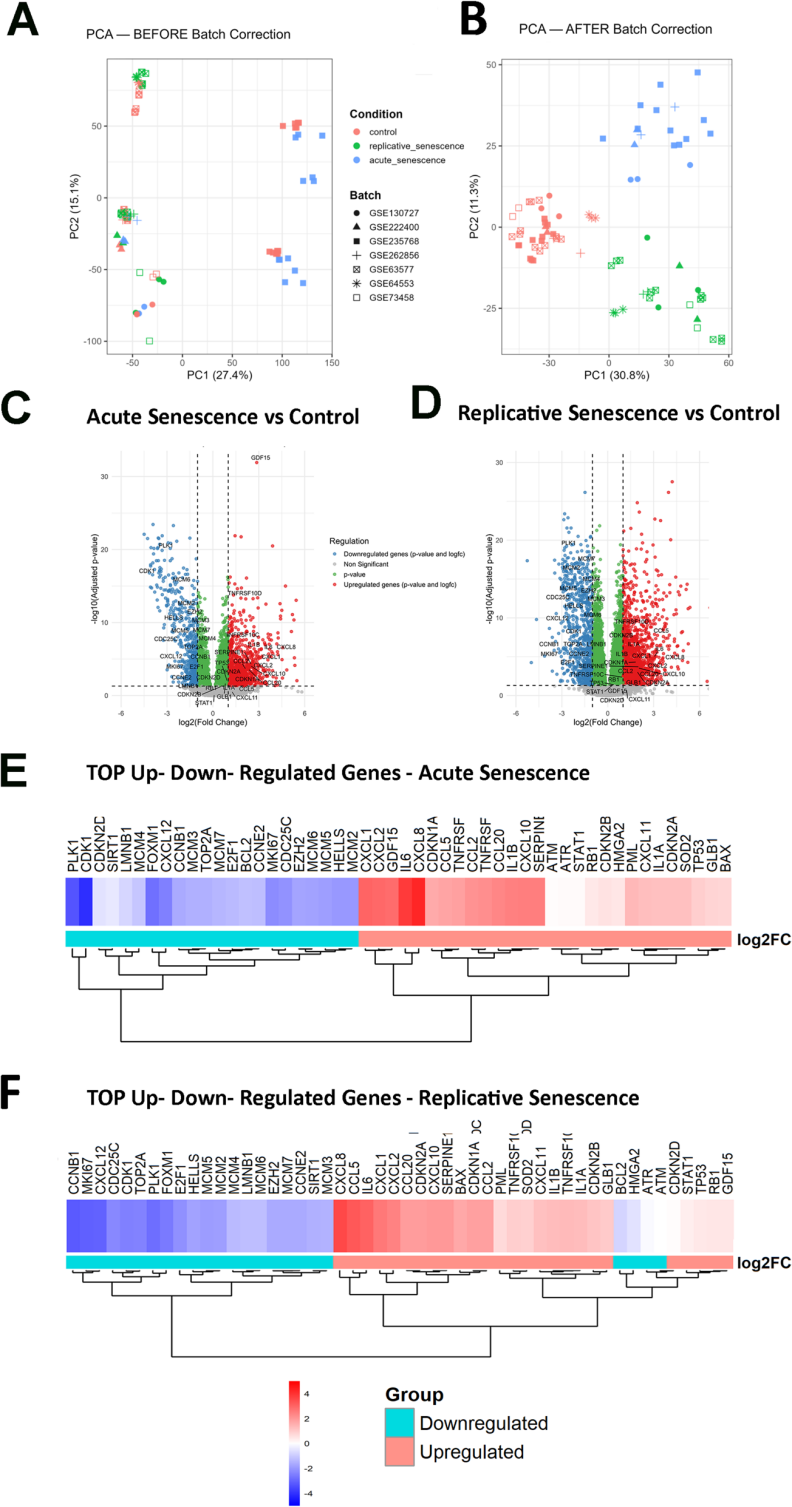
Principal component and differential expression analyses in cellular senescence. **A**, **B** Principal component analysis (PCA) of transcriptomic data before and after batch correction. Left panel shows the PCA before correction with PC1–PC2 explaining 31.3% and 16.8% of variances, highlighting the influence of batch effects. Right panel shows PCA after batch correction with PC1–PC2 explaining 17.8% and 15.2% of the variances, illustrating improved clustering aligned with biological conditions rather than technical variations. Each dot represents a sample, color-coded by condition (control, acute senescence, replicative senescence) and marked with different shapes according to the batch series it belongs to (GSE130727, GSE222400, GSE235768, GSE262856, GSE63577, GSE64553, and GSE73458). **C**, **D** Volcano plots displaying differential gene expression between acute senescence versus control (left) and replicative senescence versus control (right). Red points indicate significantly upregulated differentially expressed genes, blue points indicate significantly downregulated differentially expressed genes, green points indicate significantly differentially expressed genes with logfc < 1, and gray points represent non-significant genes. Differential expression was analyzed based on “condition,” and genes were identified as significant if they exhibited an absolute log2 fold change greater than 1 and an adjusted *p* value below 0.05, using the Benjamini–Hochberg method for false discovery rate correction. Highly reported senescence genes are highlighted in the volcano plot. **E**, **F** Heatmaps of the top significantly differentially expressed genes in acute senescence (up) and replicative senescence (down), with genes clustered based on expression patterns. Red indicates upregulation, blue indicates downregulation

To ensure that structure in the integrated dataset reflected biological condition rather than study of origin, we performed PCA on the combined transcriptomes before and after surrogate variable adjustment. Prior to correction, samples clustered primarily by GEO dataset, and PC1 and PC2 explained 27.4% and 15.1% of the variance, indicating that batch effects dominated the principal axes (Fig. 2A). After SVA, this study-driven clustering was largely attenuated and samples instead separated by biological condition, with proliferating, replicative-senescent, and acute-senescent cells forming distinct but related clusters (PC1 30.8%, PC2 11.3%; Fig. 2B). This visual shift was supported by quantitative metrics computed in PCA space. Separability of the condition increased substantially (mean silhouette 0.0636 → 0.3787), and within-condition dispersion decreased (mean within-condition distance 144.33 → 43.64), along with an improved within/between distance ratio (0.8299 → 0.5653). Consistently, within-condition kNN batch-mixing improved across all groups, with the fraction of same-batch nearest neighbors decreasing for control (0.60 → 0.27), replicative senescence (0.36 → 0.27), and acute senescence (0.68 → 0.36) as mentioned in “Data preprocessing, quality control, and normalization.” Collectively, these results indicate that harmonization reduced residual batch structure and recovered a reproducible, condition-driven transcriptomic space suitable for defining shared senescence components across distinct triggers [4].

### Senescence is defined by coordinated repression of proliferative programs and activation of inflammatory and matrix-remodeling modules

To define the transcriptional programs underlying the shared senescent state, we compared replicative- and acute-senescent fibroblasts to proliferating controls. Both conditions exhibited large-scale and highly concordant changes in gene expression, with thousands of genes either induced or repressed relative to proliferating cells. After filtering, 24,871 genes remained across both comparisons. In acute senescence, 1111 genes were upregulated and 541 were downregulated. In replicative senescence, 1594 genes were upregulated and 823 were downregulated. Despite distinct upstream triggers, the direction and functional identity of these changes were strikingly similar across senescence modalities.

Genes upregulated in both conditions were enriched for inflammatory signaling, extracellular matrix organization, and immune-modulatory functions, consistent with the establishment of a senescence-associated secretory phenotype [29]. In contrast, genes involved in DNA replication, mitotic progression, chromatin assembly, and DNA repair were uniformly repressed, reflecting permanent withdrawal from the cell cycle and large-scale nuclear reorganization [30].

Volcano plots and heatmaps illustrate this bimodal reprogramming, with inflammatory and matrix-associated genes showing coordinated induction and replication- and chromatin-associated genes showing synchronized repression in both replicative and acute senescence. This conserved transcriptional dichotomy defines the core molecular signature of the senescent state (Fig. 2). Functional categorization of characterized upregulated and downregulated genes in replicative senescence condition and acute senescence condition is reported in supplementary files (Supplementary Table 1, Supplementary Table 2, Supplementary Table 3).

### Gene Ontology reveals two conserved functional modules that define the senescent state

**Fig. 3:**
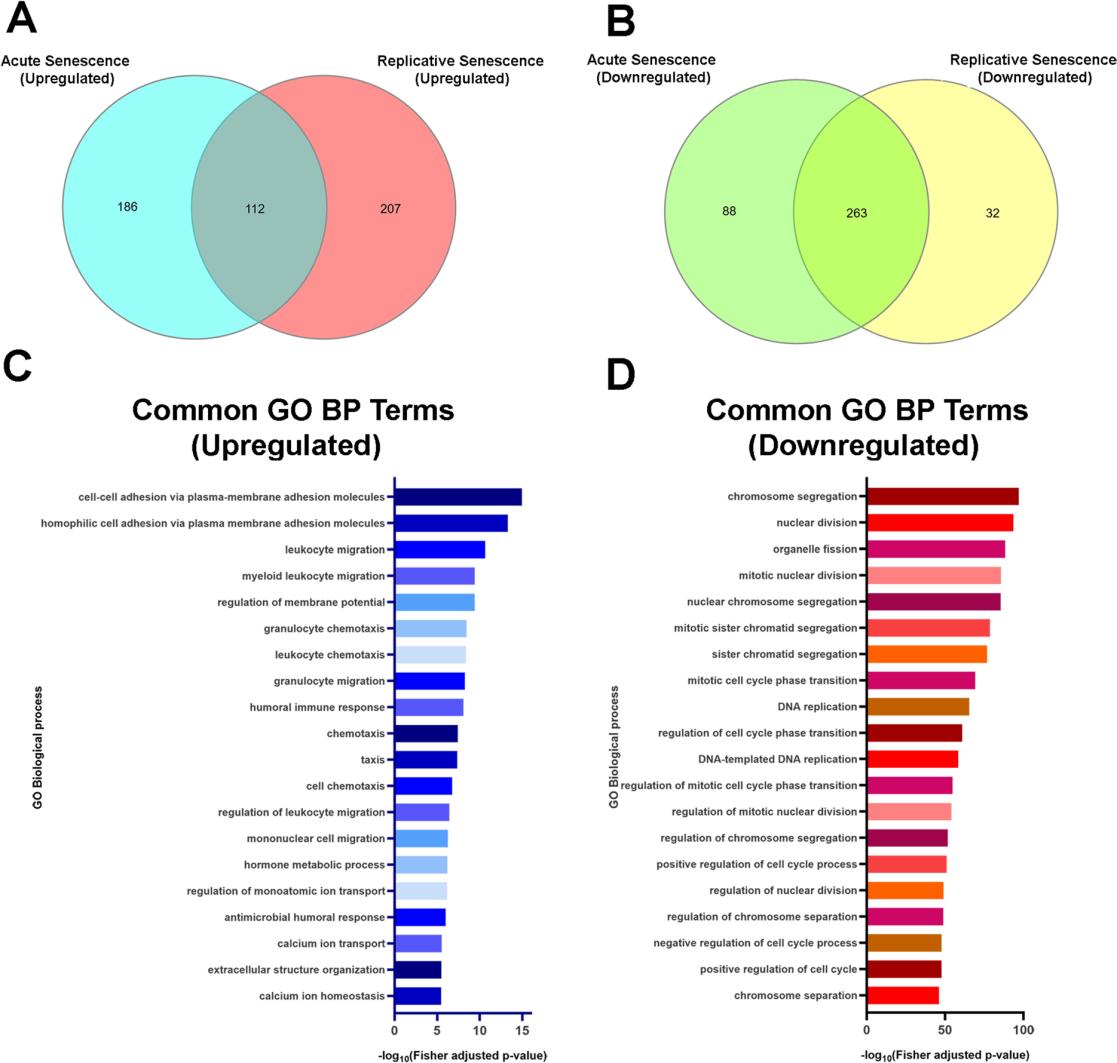
Analysis of Gene Ontology (GO) biological processes in senescent cells. **A**, **B** Venn diagrams illustrating the overlap of upregulated (left) and downregulated (right) GO biological process terms in acute and replicative senescence, highlighting specific and common ontology influenced by different senescence triggers. The left diagram shows 112 common upregulated processes, while the right diagram indicates 263 shared downregulated processes. **C**, **D** Bar graphs detailing the top 20 common GO biological process terms upregulated in senescence (left) and top 20 common GO biological process terms downregulated in senescence (right). Our analysis used the clusterProfiler, centered on terms with an adjusted *p* value (padj) less than 0.05, using the Benjamini–Hochberg procedure to control false discovery rates. All analyses utilized the human gene annotation database

To determine whether the shared senescence-associated transcriptional signature reflects a conserved functional organization, we performed Gene Ontology enrichment analysis on genes induced or repressed in replicative and acute senescence. Venn-based filtering was used to identify biological processes consistently shared across conditions (Fig. 3A) [31], followed by ranking of the top GO biological process (GO-BP) terms based on average fold change. A parallel approach was applied to downregulated genes to identify suppressed functional programs (Fig. 3B).

We identified 112 shared GO-BP terms among upregulated genes and 263 among downregulated genes (Supplementary Tables 4 and 5). Across both senescence modalities, induced genes converged on processes related to inflammatory signaling, chemotaxis, cell adhesion, and extracellular matrix organization, consistent with activation of SASP-related programs and microenvironmental remodeling [32]. In contrast, downregulated genes were strongly enriched for DNA replication, mitotic progression, chromosome segregation, and chromatin assembly, reflecting coordinated suppression of proliferative and nuclear replication-associated functions (Fig. 3C, D).

Notably, these functional patterns were conserved across both replicative and stress-induced senescence, indicating convergence toward a shared modular organization. GO analysis thus reveals a recurrent architecture in which inflammatory and extracellular processes dominate the induced program, while replication–mitosis–chromatin pathways define the repressed program [33].

Importantly, the two programs differed not only in content but also in scale. The repressed component encompassed more than twice the number of shared GO-BP terms compared to the induced component (263 vs 112, 2.35-fold difference), with consistently higher enrichment scores across both triggers. This indicates that transcriptional repression of proliferative programs is both broader and more penetrant than the induction of inflammatory responses, suggesting that transcriptional shutdown represents the structurally dominant feature of the shared senescence state. This distinction has direct implications for prioritizing candidate therapeutic targets within the senescence network.

Beyond canonical SASP-associated processes, the shared induced signature also included functional categories related to cellular excitability and signal transduction, such as calcium ion homeostasis (Fisher combined *p* = 3.40 × 10^−6^), regulation of membrane potential (*p* = 3.86 × 10^−10^), sodium ion transport (*p* = 1.67 × 10^−5^), and ERK1/ERK2 signaling (*p* = 2.07 × 10^−5^). The consistent enrichment of these terms across independent datasets and distinct senescence triggers suggests that electrophysiological and mitogenic reprogramming represent stable and underappreciated features of the senescent fibroblast state (Supplementary Table 4).

### Reactome pathway mapping confirms conserved activation of SASP/ECM signaling and repression of replication–mitotic circuits

**Fig. 4:**
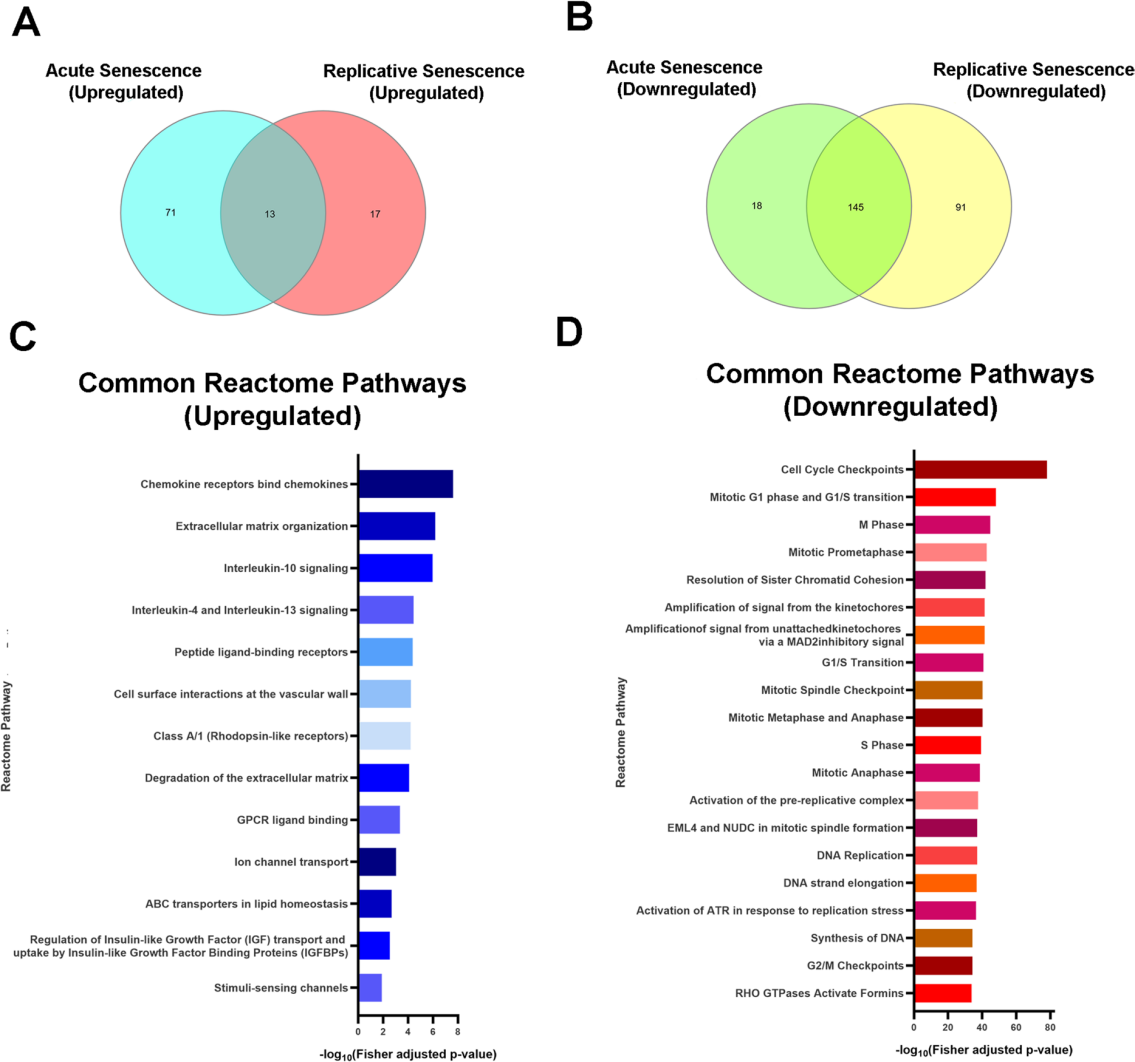
Reactome pathway analysis in senescent sells. **A, B** Venn diagrams showing the overlap of Reactome pathways that are upregulated (left) and downregulated (right) in replicative versus acute senescence. The left diagram highlights 13 pathways commonly upregulated in both types of senescence, while the right diagram shows 145 pathways commonly downregulated. **C**, **D** Bar graphs displaying the common Reactome pathways based on their significance. The left bar graph lists upregulated pathways in senescence. The right bar graph details top 20 downregulated pathways in senescence. We employed the ReactomePA packages and concentrated on terms with an adjusted *p* value (padj) under 0.05, using the Benjamini–Hochberg method to manage false discoveries. All analyses relied on the human gene annotation database

GO analysis provides a broad overview of the most significant functional categories within a dataset but does not directly identify key RNAs in the analyzed transcriptome. In contrast, Reactome analysis offers a more precise approach by highlighting critical factors based on their involvement in biological pathways [34]. To map the conserved functional modules onto defined molecular pathways, we performed Reactome enrichment analysis on genes induced or repressed in replicative and acute senescence. We identified 30 upregulated pathways in replicative senescent cells and 84 in acute senescence, with 13 pathways shared between both senescence types (Fig. 4A). The number of downregulated pathways was notably higher, with 236 in replicative senescent cells and 163 in acute senescent cells. Interestingly, 145 of these were common between the two conditions (Fig. 4B) (Supplementary Table 6, Supplementary Table 7). Across both modalities, upregulated pathways prominently involved extracellular matrix organization, cytokine and interleukin signaling, chemokine receptor pathways, and cell–surface interaction programs, consistent with sustained inflammatory communication and tissue-remodeling outputs (Fig. 4C). Conversely, downregulated pathways were dominated by DNA replication, cell cycle checkpoints, mitotic progression, and chromatin assembly–linked processes, reflecting stable proliferative exit and coordinated suppression of replication-associated nuclear programs [29, 32, 33] (Fig. 4D).

At the pathway level, the imbalance between induced and repressed programs was even more pronounced. Only 13 pathways were shared among upregulated genes across both senescence types, compared with 145 among downregulated genes (11.2-fold difference). Enrichment scores were consistently higher in the repressed set. Together with GO results, this concordant pattern across annotation systems and senescence triggers indicates that broad transcriptional silencing, rather than inflammatory activation, is the dominant structural feature of the conserved senescence program.

Notably, beyond expected replication and cell cycle pathways, two cytoskeleton-related pathways were consistently suppressed: *RHO GTPases Activate Formins* (Fisher combined *p* = 1.72 × 10^−34^; fold enrichment 7.82 and 6.47) and *RHO GTPase Effectors* (*p* = 1.31 × 10^−29^; fold enrichment 4.27 and 4.16). These pathways regulate actin dynamics, stress fiber formation, and cytoskeletal tension, linking transcriptional repression to cellular architecture and mechanotransduction. Their shared suppression across both triggers extends the senescence-associated shutdown beyond nuclear and proliferative processes to include the structural organization of the cell (Supplementary Table 7).

### Interactome embedding delineates recurrent checkpoint and replication neighborhoods in senescence

**Fig. 5:**
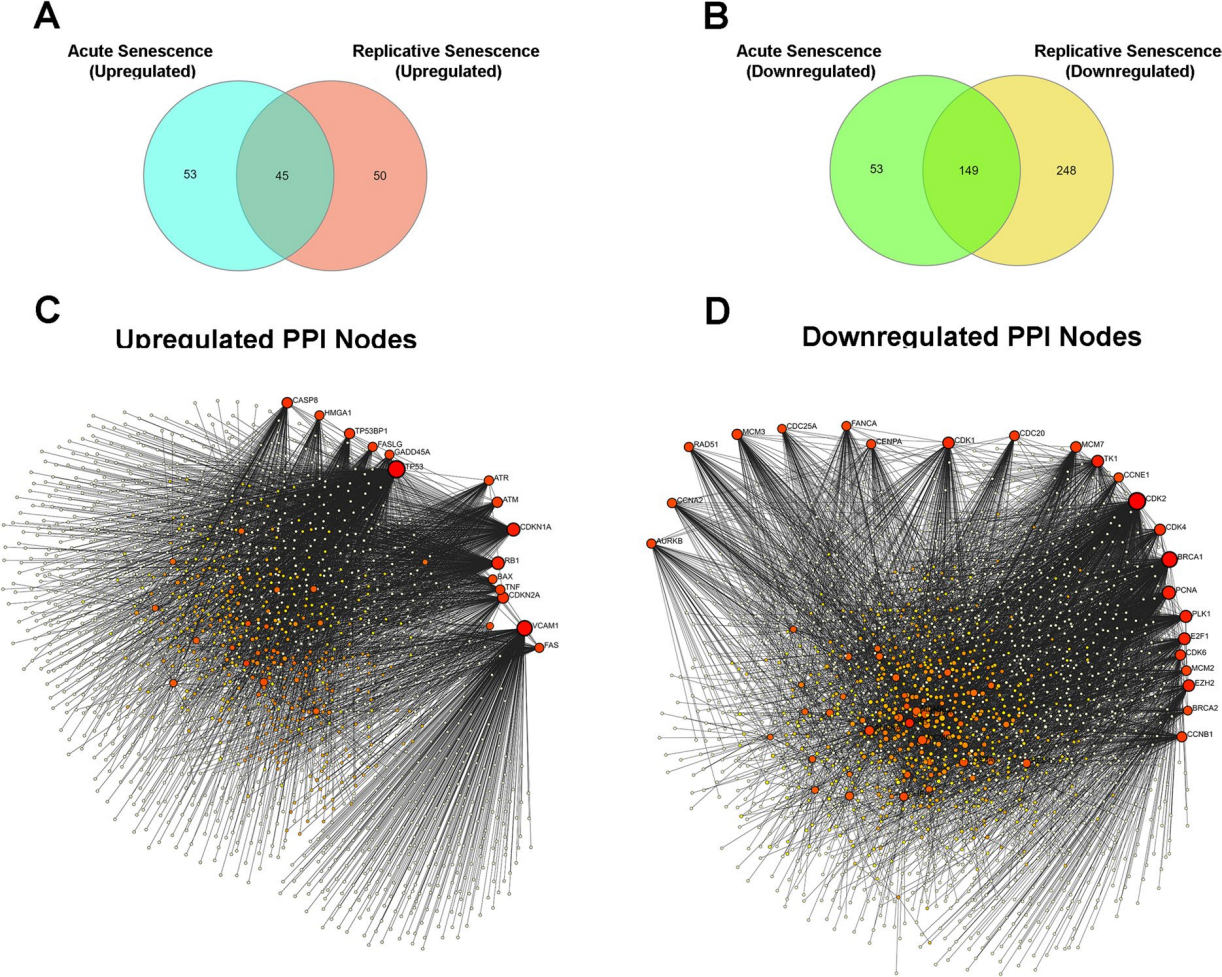
Protein–protein interaction (PPI) network analysis in senescent cells. **A**, **B** Venn diagrams displaying the overlap of PPI nodes that are upregulated (left) and downregulated (right) in acute and replicative senescence. The diagrams illustrate specific and shared regulatory genes involved in the different senescence conditions, with 45 common genes upregulated and 149 downregulated across both senescence types. **C**, **D** Network diagrams depicting the PPI nodes for upregulated genes in senescence (left) and downregulated genes in senescence (right). Diagrams are prepared utilizing the Minimum IMEx Interactome Network option, first-order option, and confidence score cutoff of 900 to simplify the networks to include only primary nodes and their direct connections. Nodes were retained if degree > 20 and betweenness > 200. These diagrams highlight the complexity of interactions within the senescence-associated cellular machinery

Shared senescence-associated genes were mapped onto a high-confidence protein–protein interaction network to transition from gene lists to network structure and identify recurrent connectors between induced and repressed programs [35]. Induced seeds comprised 95 genes in replicative senescence and 98 genes in acute senescence, including 45 shared seeds (Fig. 5A). Repressed seeds comprised 397 genes in replicative senescence and 202 genes in acute senescence, including 149 shared seeds (Fig. 5B; Supplementary Table 8).

The resulting interactome was organized into two major connectivity regions corresponding to the induced and repressed transcriptional programs. The induced network was enriched for DNA damage and checkpoint regulators (TP53, CDKN1A, CDKN2A, RB1, and ATM) together with inflammatory and extracellular matrix mediators (IL6, TNF, IL1B, CCL2, MMP9, and SPP1), placing growth-arrest control and SASP output within the same interaction space [36] (Fig. 5C). In contrast, the repressed network was dominated by replication and chromatin-associated machinery (MCM2–7, CDK1, CDK2, CCNB1, EZH2, BRCA1, LMNB1, and histone clusters), consistent with coordinated suppression of proliferative and nuclear programs [37] (Fig. 5D).

A limited set of bridging nodes connected these regions. UBC and CCND1 linked proteostasis and transcriptional control to checkpoint and cell cycle modules, while ten genes (APP, CDKN1A, EGR1, ELAVL1, GRB2, HNF4A, HSP90AA1, SP1, TP53, and UBC) occupied both induced and repressed neighborhoods simultaneously (Supplementary Table 8), defining an interface between the two programs. Among these, ELAVL1 emerges as a key post-transcriptional regulator, stabilizing both IL6 and CDKN1A transcripts and thereby coupling SASP activity with growth arrest.

Within the repressed network, PARP1 co-localized with BRCA1, BRCA2, and RAD51, whereas ATM and TP53 remained in the induced network. This configuration indicates sustained DNA damage signaling alongside reduced repair capacity, a combination that prevents damage resolution and reinforces checkpoint activation.

Overall, the interactome reveals a structurally coupled organization in which induced and repressed programs are interconnected through a compact set of shared nodes, providing a network-level view of senescence while avoiding assumptions of causal directionality or dynamic maintenance [38, 39].

## Discussion

### Cross-study integration reveals a conserved architecture of senescence

This study establishes a cross-study transcriptomic integration of human fibroblast senescence, supported by rigorous normalization and batch-mixing diagnostics demonstrating that biological condition, rather than study-of-origin, drives the observed structure. Within this validated space, we identify a recurrent senescence component shared between replicative and acute stress–induced conditions. Importantly, the novelty of this work does not lie in the identification of individual senescence-associated genes but in demonstrating that these elements consistently organize into a conserved and reproducible regulatory architecture across independent datasets and distinct inducing triggers.

These findings shift the interpretation of cellular senescence from a collection of differentially expressed genes or enriched pathways to a structured systems-level state. Across independent datasets and distinct triggers, senescent fibroblasts converge on a shared organization composed of tightly coupled functional modules, indicating that senescence represents a constrained and reproducible configuration of cellular behavior rather than a trigger-specific transcriptional outcome.

### Challenges in the use of transcriptomic datasets to identify mechanisms governing cellular processes

Previous transcriptomic studies of senescence have largely focused on single triggers and enrichment-based analyses, which identify altered biological processes but do not resolve their structural relationships. By integrating Gene Ontology, Reactome pathway mapping, and interactome embedding within a unified analytical framework, this study reveals higher-order properties of the senescence program, including cross-trigger recurrence, network coupling, and the presence of shared interface nodes that are not detectable through enrichment analysis alone [12, 40, 41].

### Coupled induction and repression define the senescence program

Across both replicative and stress-induced senescence, induced genes consistently mapped to inflammatory, SASP, and extracellular matrix programs, while repressed genes converged on replication, mitotic, and chromatin-associated functions. Crucially, the interactome layer demonstrates that these programs are not independent but physically connected within a shared network architecture. A subset of genes, including APP, CDKN1A, EGR1, ELAVL1, GRB2, HNF4A, HSP90AA1, SP1, TP53, and UBC, occupy positions at the interface of induced and repressed neighborhoods, defining a structural coupling between growth arrest and inflammatory output that cannot be resolved by enrichment analysis alone [42]. These network-defined hubs may represent candidate targets for senomorphic or senolytic interventions.

### Dominance of transcriptional repression and emergence of non-canonical programs

Integration of functional and pathway-level analyses reveals that transcriptional repression represents the dominant component of the shared senescence program. The repressed network is broader and more strongly enriched than the induced one across both senescence triggers, indicating that shutdown of proliferative and chromatin-associated processes is a primary structural feature of senescence.

In addition to canonical SASP-associated programs, the shared induced signature includes consistent upregulation of calcium ion homeostasis, membrane potential regulation, and ERK1/ERK2 signaling, suggesting a previously underappreciated layer of electrophysiological and mitogenic reprogramming. Furthermore, suppression of RHO GTPase–Formin signaling extends the shared shutdown program to cytoskeletal organization, linking transcriptional regulation to cellular mechanics and structural remodeling.

### Key regulatory hubs define the senescence backbone

Integrated GO, Reactome, and PPI analyses show that cellular senescence is not a random damage response but a coordinated systems-level program. Its stability comes from a small set of interconnected modules that collectively enforce cell cycle arrest, sustain DNA damage signaling, reshape chromatin organization, and maintain metabolic and inflammatory activity. Below, we summarize the main functional circuits that make up this self-sustaining senescence network.

#### Cell cycle arrest and checkpoint enforcement

Senescence is characterized by a stable cell cycle blockade driven by checkpoint activation and suppression of replication machinery [43]. Core regulators of G1/S arrest, including TP53, CDKN1A, CDKN2A, RB1, ATM, and CCND1, are consistently upregulated. The TP53–p21 axis enforces cell cycle exit through inhibition of cyclin-dependent kinases, while RB1 mediates chromatin-associated repression of E2F target genes [44]. Notably, CCND1 emerges as a high-centrality connector linking checkpoint, chromatin, and inflammatory modules.

In parallel, key drivers of cell cycle progression (CDK1, CDK2, CCNA2, CCNB1, CDC20, PLK1, and MCM2–7) are strongly downregulated, indicating coordinated silencing of mitotic and replication programs. Loss of mitotic checkpoint regulators such as AURKA, AURKB, BUB1B, and TRIP13 further reinforces the irreversible nature of proliferative arrest [43].

#### DNA damage response and repair pathways

Senescent cells maintain persistent activation of the ATM–TP53 axis, sustaining DNA damage signaling despite reduced repair capacity. Elevated ATM, TP53, and CDKN1A reflect ongoing checkpoint activity, while TNFRSF10D contributes to apoptosis resistance and prolonged survival [45, 46]. Conversely, downregulation of BRCA1, BARD1, KIAA0101, RAD51, and TOP2A indicates suppression of homologous recombination and replication-associated repair processes [37]. A key structural feature is the co-occurrence of PARP1 within the repressed network alongside BRCA1, BRCA2, and RAD51, while ATM and TP53 remain induced. This configuration reflects a self-reinforcing state in which persistent damage signaling is coupled to impaired repair, maintaining checkpoint activation and stabilizing senescence across triggers.

#### Chromatin remodeling and epigenetic reorganization

Large-scale chromatin reorganization is a defining feature of senescence, leading to stable repression of proliferation-associated genes. Downregulation of EZH2, LMNB1, and histone cluster genes (HIST1H/HIST2H families) reflects polycomb-mediated repression and disruption of nuclear lamina integrity, consistent with senescence-associated heterochromatin foci (SAHF) formation. Loss of EZH2 reduces H3K27 methylation and transcriptional plasticity, while LMNB1 depletion weakens nuclear architecture. At the same time, induced genes such as SPP1, FOXA1, and HNF4A link chromatin remodeling to inflammatory and metabolic pathways, suggesting that nuclear reorganization is functionally integrated with SASP regulation [47, 48].

#### Inflammatory and SASP pathways

SASP-associated genes form a dominant inflammatory module that contributes to stabilization of senescence and remodeling of the tissue microenvironment. Key nodes, including IL6, TNF, IL1B, CCL2, MMP9, PLAUR, and SPP1, are directly connected to checkpoint regulators such as TP53 and CDKN1A [49]. indicating coordinated regulation rather than a secondary consequence of growth arrest. Downregulation of SOCS1, a negative regulator of cytokine signaling, further amplifies NF-κB-driven transcription, sustaining chronic paracrine activity characteristic of senescent cells [50].

#### Metabolic and proteostatic adaptation

Senescent cells undergo extensive metabolic and proteostatic reprogramming to maintain viability under persistent stress. Upregulated hubs such as APP, HNF4A, FOXA1, ELAVL1, UBC, and CCND1 form an interconnected module integrating metabolism, RNA stability, and protein turnover [51]. ELAVL1 plays a central role by stabilizing both inflammatory transcripts (e.g., IL6, TNF) and the cell cycle inhibitor CDKN1A, positioning it at the interface of SASP maintenance and growth arrest. Its consistent localization within both induced and repressed interaction neighborhoods reveals a post-transcriptional coordination layer not detectable through enrichment analysis alone. UBC, the most central node in the network, highlights the importance of ubiquitin-mediated proteostasis in buffering protein turnover under sustained stress. In parallel, APP and HNF4A connect lipid metabolism and mitochondrial function to chromatin accessibility, supporting a metabolically active yet non-proliferative cellular state [52]. Together, these features indicate that long-term survival of senescent cells depends not only on transcriptional arrest but also on coordinated stabilization of metabolic and proteostatic circuits.

From a systems perspective, this convergence is consistent with the emergence of an attractor-like state, in which cellular behavior is constrained by a stable network configuration rather than by transient upstream signals. Senescence thus represents a low-dimensional, self-stabilizing state defined by the coupling of checkpoint signaling, chromatin organization, and inflammatory output [53, 54].

### Scope, generalizability, and future extensions of the senescence network model

A limitation of the present study is that it focuses on human fibroblasts, reflecting the fact that this cell type currently provides the most extensive and best-annotated transcriptomic coverage of both replicative and stress-induced senescence. However, this constraint also represents a strength: Fibroblasts are the prototypical senescence model and the primary contributors to tissue-level SASP and extracellular matrix remodeling in vivo [3, 55]. As such, defining the regulatory architecture of fibroblast senescence provides a reference framework against which other lineages can be compared.

The systems-level architecture identified here is not expected to be fibroblast-specific. Rather, it likely represents a conserved network logic by which diverse cell types stabilize a senescent state, even if the specific SASP components and metabolic adaptations differ. Extending this integrative approach to epithelial, endothelial, immune, and stem cell populations using emerging multi-omic and single-cell datasets will allow direct testing of the universality of this architecture and identification of lineage-specific modifications. Thus, the present work establishes a foundational map of senescence maintenance circuits that can be iteratively refined as higher-resolution and multi-lineage datasets become available.

## Conclusion

Cellular senescence emerges here as a structured, multi-layered network state rather than a loose collection of differentially enriched processes. By integrating seven public human fibroblast RNA-seq datasets spanning replicative exhaustion and acute stress–induced senescence, we demonstrate that distinct triggers converge on a conserved regulatory architecture stabilizing the senescent phenotype. This architecture couples checkpoint enforcement and chromatin-linked repression of proliferative programs with sustained SASP and extracellular matrix activity.

Within this framework, a recurrent cross-trigger component is consistently recovered across transcriptomic, functional, pathway, and interactomic levels. Interactome embedding further reveals that induced and repressed programs are not independent but organized within a shared connectivity network, highlighting bridging nodes that define the structural backbone of senescence and provide prioritization candidates not identifiable through enrichment analysis alone.

Beyond canonical senescence biology, this integration identifies conserved upregulation of calcium signaling, membrane potential regulation, and ERK1/ERK2 pathways, alongside repression of RHO GTPase–Formin signaling, extending the shared program to electrophysiological and cytoskeletal dimensions. At the network level, a subset of genes occupies both induced and repressed neighborhoods, with ELAVL1 coordinating SASP and growth arrest post-transcriptionally, and the co-occurrence of suppressed PARP1 with active ATM defining a self-reinforcing damage configuration not previously described in human fibroblasts.

Together, these results establish a quantitative cross-study reference for senescence and define a structured network framework for prioritizing candidate nodes for senomorphic or senolytic intervention in aging and age-related diseases.

## Supplementary Information

Below is the link to the electronic supplementary material.

ESM 1 (PDF 417 KB)

ESM 2 (PDF 2.66 MB)

ESM 3 (DOCX 95.0 KB)

## Data Availability

The code used for data processing and analysis is openly available at the following GitHub repository: https://github.com/Mohd-Shahzaib/Senescence-Transcriptome-MetaAnalysis./tree/main/Data_of_DEG/DESeq2_SVA_pipeline. All datasets generated and analyzed during this study are included in the published article and its supplementary materials. Additional information is available from the corresponding author upon reasonable request.

## Abbreviations

BP: Biological process
DEGs: Differentially expressed genes
FDR: False discovery rate
GEO: Gene Expression Omnibus
GO: Gene Ontology
GO:BP: Gene Ontology Biological Process
PCA: Principal component analysis
PDL: Population doubling level
PPI: Protein–protein interaction
RNA-seq: RNA sequencing
SAHF: Senescence-associated heterochromatin foci
SASP: Senescence-associated secretory phenotype
scRNA-Seq: Single-cell RNA sequencing
SVA: Surrogate Variable Analysis
VST: Variance-stabilizing transformation
