# Supplementary material for: A conserved regulatory architecture stabilizes cellular senescence across distinct triggers in human fibroblasts
Source: GeroScience. 2026 May 7;48(3):3511–29. doi: 10.1007/s11357-026-02297-6 (PMC13356186; doi:10.1007/s11357-026-02297-6)
Supplement: Supplementary file 1 — (PDF 417 KB) [file 11357_2026_2297_MOESM1_ESM.pdf]

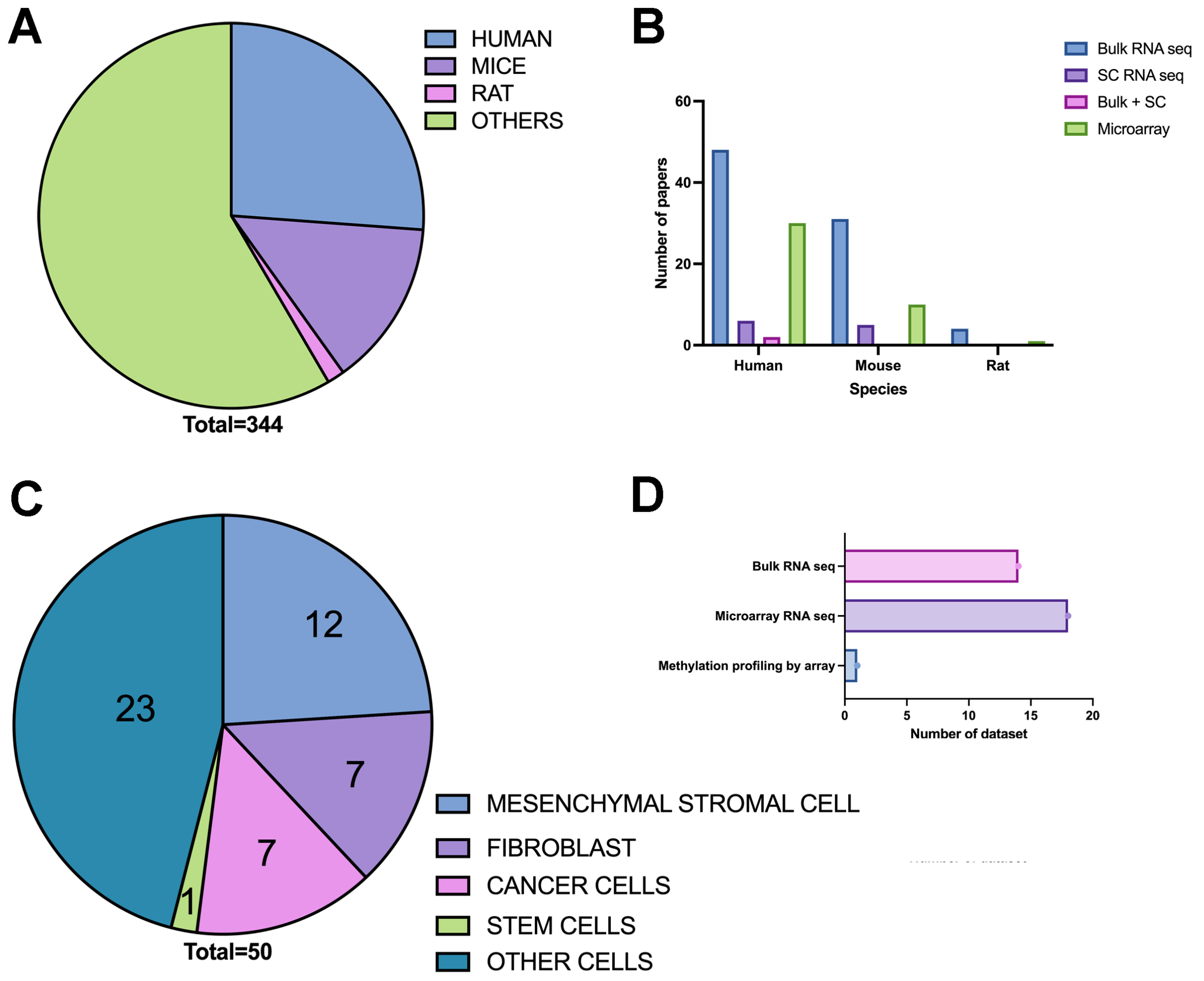

Supplementary Figure 1. Selection and composition of transcriptomic datasets used for senescence meta-analysis

A Pie chart showing the species distribution of 344 PubMed records initially identified in the literature survey using senescence-related transcriptomic search terms. Human studies constitute 26.2% of the total, followed by mouse (15.4%) and other species (58.4%).

B Bar graph indicating the number and type of transcriptomic studies available for each species, distinguishing between bulk RNA-seq, single-cell RNA-seq (scRNA-seq), combined bulk + scRNA-seq, and microarray-based datasets. Human datasets dominate the RNA-seq category and provide the primary source of transcriptomic material suitable for cross-study integration.

C Pie chart summarizing the cellular origin of 50 human bulk and combined RNA-seq datasets identified from the literature, showing the distribution across mesenchymal stromal cells, fibroblasts, cancer cells, stem cells, and other cell types. Fibroblasts represent a major fraction of the available senescence-focused transcriptomic datasets.

D Bar graph showing the number of publicly available GEO datasets identified by platform when querying senescence-related human studies, including bulk RNA-seq, microarray-based RNA-seq,

and methylation profiling by array. Only bulk RNA-seq datasets fulfilling strict inclusion criteria were retained for downstream integrative analysis.
