# Supplementary material for: A conserved regulatory architecture stabilizes cellular senescence across distinct triggers in human fibroblasts
Source: GeroScience. 2026 May 7;48(3):3511–29. doi: 10.1007/s11357-026-02297-6 (PMC13356186; doi:10.1007/s11357-026-02297-6)
Supplement: Supplementary file 2 — (PDF 2.66 MB) [file 11357_2026_2297_MOESM2_ESM.pdf]

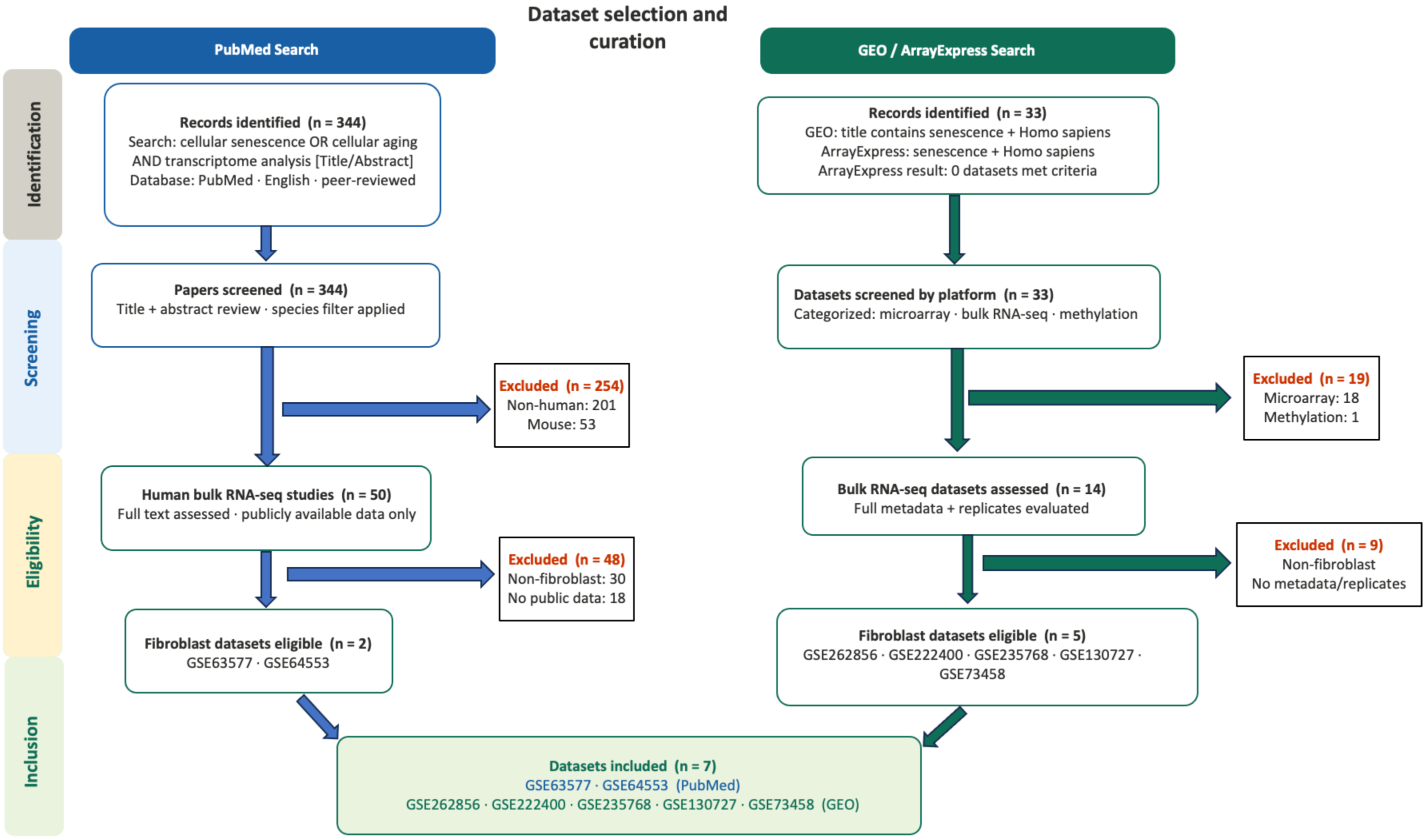

Supplementary Figure 2. Dataset selection and curation workflow.

Systematic identification and filtering of publicly available transcriptomic datasets from PubMed and GEO/ArrayExpress repositories. Studies were screened and excluded based on predefined criteria (non-human, non-fibroblast, insufficient metadata or replicates). The final dataset includes seven independent human fibroblast RNA-seq datasets representing replicative and stress-induced senescence.
