# Supplementary material for: A conserved regulatory architecture stabilizes cellular senescence across distinct triggers in human fibroblasts
Source: GeroScience. 2026 May 7;48(3):3511–29. doi: 10.1007/s11357-026-02297-6 (PMC13356186; doi:10.1007/s11357-026-02297-6)
Supplement: Supplementary file 3 — (DOCX 95.0 KB) [file 11357_2026_2297_MOESM3_ESM.docx]

**Table 1: Functional Categorization of Characterized Upregulated and Downregulated Genes in Acute senescence Condition**

| **Category** | **Gene Symbols** | **Function** | **Regulation** |
| --- | --- | --- | --- |
| Non-Coding RNAs (ncRNAs) | SPRY1, LINC00158, TSPEAR-AS1, LINC01260, HEXA-AS1, ARHGAP5-AS1, LINC00525, LINC00334, TSPEAR-AS2, A2M-AS1, LINC02691, LINC00519, LINC00504, IGFBP7-AS1, PDCD4-AS1, LINC01588, LINC00640, LINC03034, LINC00908, LINC00663, LINC01091, LINC01622, C8ORF34-AS1, KRTAP5-AS1, LINC01252, FMNL1-DT, SOX2-OT, LINC00965, LINC02716, ZNF582-DT, LINC00222, NSMCE1-DT, ZC3H18-AS1, TOB1-AS1, KCNJ2-AS1, LINC02864, LINC02817, LINC00243, LINC02902, FOSL2-AS1 | Guide rRNA modifications and post-transcriptional control; shape immune and apoptotic responses. | Upregulated |
| Immune Response and Inflammation | BST2, C3, C4BPA, C5AR1, C9, CCR1, ACKR2, CMKLR1, CX3CR1, DOCK3, XCR1, CXCL1, CXCL2, CXCL3, CFHR1, IL1A, IL1B, IL6, CXCL8, IL12A, CXCL10, CCL2, CCL5, CCL7, CCL11, CCL13, CCL20, CCL25, CXCL5, CX3CL1, XK, CXCL14, CCL26, CFHR3, ACKR4, IL20RB, IL17RB, ACKR3, DOCK8, PELATON | Cytokines, chemokines, antigen presentation; leukocyte recruitment and inflammatory signaling. | Upregulated |
| Signal Transduction and Cell Communication | AK4, ADGRB3, LDLRAD4, CAMK2B, CCKAR, CDKN1A, CDKN2A, CDKN2B, CHRNA7, CNTFR, CSF2RA, CSF2RB, CSF3R, DAPK1, EGF, CELSR3, ADGRE1, EPHA8, ERBB4, FCGR1A, FCGR2A, FOLR1, FOLR3, FRK, GABRA2, GABRR2, GHR, GLRB, NPBWR1, GRIN1, GRIN2B, HTR1D, ITK, KLRD1, LEPR, LRP2, NR3C2, MSR1, MST1R, NTRK3 | Receptor/kinase/adapter circuits transmitting extracellular cues to transcriptional programs. | Upregulated |
| Metabolism and Cellular Homeostasis | ATP1A3, ATP1B2, ATP6V0A1, ATP6, ATP8, SLCO1A2, ATP8A1, NPC2, TXNIP, SLCO2B1, CNNM1, NPC1L1, ATP6V0A4, FLVCR2, ATP8B4, ATP13A4, PRXL2A, NKAIN2, ATP6V0D2 | Detoxification, redox balance, transporters and pumps; ion and nutrient homeostasis. | Upregulated |
| Structural and Cytoskeletal Components | ASTN1, CNTN1, COL4A3, COL4A4, COL19A1, DNAH8, DMTN, ICAM1, L1CAM, MAP6, MAPT, MGP, MMP1, MMP3, MMP9, MMP10, MMP12, MMP15, NRCAM, COLQ, HRK, MAP7, INA, CYTIP, FRY, CHL1, FILIP1L, DNAAF11, CADM1, MXRA5, ATRNL1, CNTNAP2, ERC2, CYFIP2, CNTN5, SDK2, DNAH3, PBXIP1, AMIGO1, CADM3 | Cytoskeleton and matrix components that support shape, transport and mechanics. | Upregulated |
| Neurotransmission and Nervous System Development | GPR27, NPY, NPAS1, SYT1, GPR65, SYNGR2, SYNGR1, SNAP91, LRRN2, RIMS1, NFASC, NSG1, GPR78, GPR132, NSG2, SYT17, LRRN3, NPAS3, LYNX1, SYT3, RIMS4, GPR155, SYCP2L, SYPL2, INSYN1, CDNF | Synaptic transmission, ion channels, GPCRs and neuronal excitability. | Upregulated |
| Apoptosis and Cell Death | BAX, ELAPOR1, PERP | Effectors and regulators of programmed cell death and survival balance. | Upregulated |
| Transcription and Gene Regulation | ELF3, H1-2, HLF, NAP1L2, NAP1L3, H2AC6, H2BC4, H4C8, GTF2A1L, KLF8, TFEC, ETV7, H2BC12L, HES2, MAML3, H2BC12, KLF14 | Transcription factors and chromatin modulators governing gene expression programs. | Upregulated |
| Cell Cycle Regulation and Proliferation | CCPG1, PPM1H, PPM1L, INSC, DNAAF4-CCPG1 | Cyclins, CDKs and checkpoints that drive cell cycle transitions. | Upregulated |
| Miscellaneous Categories | A2M, NAT2, ABCA3, ACHE, ACR, ADCY5, ADH1B, ADRA2C, AGT, ALB, ALDH3A1, ABCD2, ALDOB, AKR1B1, ALOX15B, ALPP, AMPD3, AMY1A, AMY1B, AMY1C, AMY2A, AMY2B, ANK1, ANK2, AOC2, APLP1, APOD, ABCC6, AREG, ARHGDIG, PHOX2A, ARRB1, ART3, ASAH1, ASPA, KIF1A, AZU1, BFSP1, BHMT, BMP2 | Stress responses, hormone/peptide signaling and other uncategorized functions. | Upregulated |
| Non-Coding RNAs (ncRNAs) | SNORD125, ARHGAP44-AS1, TMPO-AS1, LINC01960, PCBP3-AS1, PCOLCE-AS1, HMGA2-AS1, LINC01415, MIR1178, MIR1244-1, PCNA-AS1, MIR1244-3, MIR1244-2, MIR3153, LINC00673, MIR3917, PBX1-AS1, CFAP58-DT, ELOVL2-AS1, JARID2-AS1, PRC1-AS1, LINC00707, LCT-AS1, LINC03014, DLG3-AS1, NAV2-AS2, SGO1-AS1, THOC7-AS1, DIAPH3-AS1, ALMS1-IT1, APOBEC3B-AS1, LINC01910, EZR-AS1, LINC03020, DEPDC1-AS1, TROAP-AS1, EP300-AS1, ITPRIP-AS1, HMMR-AS1, GRK5-IT1 | Guide rRNA modifications and post-transcriptional control; shape immune and apoptotic responses. | Downregulated |
| Immune Response and Inflammation | SPAG5, FANCA, FANCD2, FANCB, FANCG, C1QL4, IL5, IL11, LY6K, IL17RD, FANCI, CXCL12, XRCC2, SOCS1, PRC1, IL33, CRLF1 | Cytokines, chemokines, antigen presentation; leukocyte recruitment and inflammatory signaling. | Downregulated |
| Signal Transduction and Cell Communication | NR2E3, CDK2, CDK4, CALCRL, CDK6, CDKN2C, CDKN3, ERN2, PLK4, CHEK1, CIT, CHRNA3, CHRNA5, CKS1B, CKS2, DCC, DTYMK, EPHB1, EPHB6, ERBB3, F2RL1, FES, FGFR4, P2RX2, LCORL, GABRA4, GABRB1, RGS17, HCAR1, GLRA2, LPAR4, MCHR1, TRBV13, SCARA5, TRBJ2-3, TRBC1, TRAC, PKN3, HMMR, HTR1B | Receptor/kinase/adapter circuits transmitting extracellular cues to transcriptional programs. | Downregulated |
| Structural and Cytoskeletal Components | CNTNAP3C, TRAIP, ZWINT, OIP5, CKAP2L, DES, TPX2, ARC, MATCAP2, ASPM, NEFH, COL5A3, PCOLCE, ANLN, PIMREG, HHIP, SPTB, CNTNAP3B, BRIP1, TICRR, SNCAIP | Cytoskeleton and matrix components that support shape, transport and mechanics. | Downregulated |
| Neurotransmission and Nervous System Development | NGEF, NPAS4, GPR4, NPW, GRIA4, NNAT, NPTX2, NRGN, GPR85, NDNF, NETO2, NETO1, SYTL1, NAV2 | Synaptic transmission, ion channels, GPCRs and neuronal excitability. | Downregulated |
| Apoptosis and Cell Death | DDIAS, BLID, ECSCR | Effectors and regulators of programmed cell death and survival balance. | Downregulated |
| Transcription and Gene Regulation | E2F1, E2F2, E2F3, EYA2, EZH2, FOXM1, AFF2, HEY1, AUTS2, H1-5, H2AX, H2AZ1, HES1, MYB, BCL11A, ASF1B, TBX20, RBL1, SIM2, SNAI1, SOX11, TCF7, TCF19, TFAM, TFAP4, E2F8, CDT1, CHAF1B, H2AC14, H2AC16, H2AC4, H2BC9, H3C3, H3C11, H3C8, H3C12, H3C2, H4C3, H4C2, L3MBTL4 | Transcription factors and chromatin modulators governing gene expression programs. | Downregulated |
| DNA Replication and Repair | MMS22L, GMNN, PCNA, MCM10, RFC2, RFC3, RFC4, RPA3, BRCA1, BRCA2, DSCC1, CDC45, MCM8 | Replication origin licensing and DNA damage response/repair modules. | Downregulated |
| Cell Cycle Regulation and Proliferation | HUS1B, SKA1, SKA3, ZYG11A, G0S2, NUSAP1, GTSE1, ERCC6L, NCAPG2, SHCBP1, CCNF, PTTG1, ESPL1, CDC20 | Cyclins, CDKs and checkpoints that drive cell cycle transitions. | Downregulated |
| Miscellaneous Categories | C8ORF88, ZNF730, TSTD1, PP12613, TROAP, SMC4, PHOSPHO2-KLHL23, ZNF670-ZNF695, ARHGAP19-SLIT1, GS1-24F4.2, GPC6, FSBP, TSPAN2, KIF20A, RASGRP1, EMSLR, RNVU1-6, DDX39A, HOTS, TUBB3, SCML2, NDC80, TACC3, FST, SEMA6B, NEBL, RNASEH2A, SMILR, CH507-145C22.1, CENPA, MAB21L2, PAICS, CENPE, CENPF, POSTN, RAD51AP1, BUB1B-PAK6, POLQ, WDR4, MTHFD2 | Stress responses, hormone/peptide signaling and other uncategorized functions. | Downregulated |

**Table 2: Functional Categorization of Characterized Upregulated and Downregulated Genes in Acute senescence Condition**

| **Category** | **Gene Symbols** | **Function** | **Regulation** |
| --- | --- | --- | --- |
| Non-Coding RNAs (ncRNAs) | LINC01705, LINC00520, PPP1R14B-AS1, MIR3659HG, LINC02154, ZNF567-DT, LINC01173, HNRNPUL2-BSCL2, IER3-AS1, TOB1-AS1, DLGAP1-AS1, VOPP1-DT, LINC02863, LINC02029, LINC00663, PAPPA-AS1, RNU1-4, MIR147B, LINC01655, LINC01588, AFF1-AS1, HFE-AS1, LINC02340, LINC03011, PLA2G4C-AS1, INKA2-AS1, PPP1R26-AS1, DYRK3-AS1, RNU1-1, ACTR3-AS1, C1QTNF1-AS1, RNU1-2, LINC02621, UNC5B-AS1, RNU1-3, LINC-PINT, STXBP5-AS1, LINC02818, LINC02908, MIR3936HG | Guide rRNA modifications and post-transcriptional control; shape immune and apoptotic responses. | Upregulated |
| Immune Response and Inflammation | CCL26, CXCL5, CCL20, CCL2, CXCL8, IL6, IL1B, CXCL2, IL13RA2, A1CF, CXCL1, XCR1, IL11, C8G, IL24, CXCL6, IRAK2, CCR7, IL33, C5AR2, IL1RL1, C3, IL1RN, TAFA3, C9, ACKR2, IL1RL2, CCL5, IL1A, CCL13, IL36RN, CCL7, CXCL10, CXCL3 | Cytokines, chemokines, antigen presentation; leukocyte recruitment and inflammatory signaling. | Upregulated |
| Signal Transduction and Cell Communication | TNFRSF10A, BCAP31, LRPAP1, TNFRSF10D, P2RX6, NRIP3, AGTRAP, STK16, MAPK13, TNFRSF10C, ADGRV1, PHKG2, DGKA, PTPRN, FOLR3, FCGR1A, CDKN2A, REEP2, IRAG2, GRIN3B, RGS18, PFKFB4, STYK1, SSTR2, MAP3K19, P2RY2, GRM8, LRP2, PIK3C2G, GABBR2, GRB14, CABYR, CKM, OPRD1, CHRNA9, F11R, NYAP2, TRPM8, PTPRC, EGF | Receptor/kinase/adapter circuits transmitting extracellular cues to transcriptional programs. | Upregulated |
| Metabolism and Cellular Homeostasis | ATP6V0A1, TANGO2, FDXR, ATP6V0D2, ATP6V1A, ATP2B2, NPC1, ATP8A1, ATP6V0A4, ATP13A4, SLCO2B1, ATP8B4, NKAIN3 | Detoxification, redox balance, transporters and pumps; ion and nutrient homeostasis. | Upregulated |
| Structural and Cytoskeletal Components | DNAI3, CYFIP2, COL10A1, MAP1A, MMP12, ASTN2, MMP3, MMP1, ICAM1, MRNIP, COL7A1, MAPRE3, DNAH3, MAP7, TNIP3, COL17A1, NECTIN4, PCLO, DNAH8, XIRP2, INKA2, COL19A1, COL2A1, AXDND1, COL28A1, ABLIM2, MMP10, MADCAM1, CNTNAP5, HOOK1, DNAH12, NEFM, CEACAM1, EPCAM, DNAH9, DNAH6, RASIP1, LNC-LBCS, SDK2, MLIP | Cytoskeleton and matrix components that support shape, transport and mechanics. | Upregulated |
| Neurotransmission and Nervous System Development | GDNF, SYN2, LYNX1, CDNF, NSG2, GPR35, SNAP91, GPR158, GRIA2, GPR87, ESYT3, GPR65, NFASC, SV2C, GPR55, SYN3, SYT12, SYT17 | Synaptic transmission, ion channels, GPCRs and neuronal excitability. | Upregulated |
| Apoptosis and Cell Death | ELAPOR1, TP53AIP1 | Effectors and regulators of programmed cell death and survival balance. | Upregulated |
| Transcription and Gene Regulation | H2AC19, H2AC18, HES2, MAFF, H2BC12, H3C4, H2BC21, NAP1L2, H1-2, H2BC5, TFEC, MAFK, MLLT11, STAT4, H2AC6, H2BC8, H2AC8, GRHL3, ELF3, ATF3, H2BC15, H2BC4, H4C8, HEYL, TFCP2L1, MAFA | Transcription factors and chromatin modulators governing gene expression programs. | Upregulated |
| DNA Replication and Repair | TERB1 | Replication origin licensing and DNA damage response/repair modules. | Upregulated |
| Cell Cycle Regulation and Proliferation | PPM1J, CCNO, HIGD2B | Cyclins, CDKs and checkpoints that drive cell cycle transitions. | Upregulated |
| Miscellaneous Categories | GDF15, RRM2B, CES3, ACER2, BSCL2, CYB5R1, HSPA4L, KIF1A, NIPAL3, TUBA4A, RRAD, AKR1B1, DYNC1I1, TFPI2, DEDD2, PDE4C, DYSF, TSPYL2, UBC, PPP2R2C, FAM219A, SMIM3, TSPAN13, DYNLT4, SAT1, TMEM179, ATG4A, STC1, MYH14, TMEM217, MAN2B1, TAF13, CTSD, GLA, ADAM15, TMEM161A, PLEKHB2, C15ORF48, HSPA1A, MBOAT7 | Stress responses, hormone/peptide signaling and other uncategorized functions. | Upregulated |
| Non-Coding RNAs (ncRNAs) | TMPO-AS1, MIR1244-1, MIR1244-2, MIR3917, TOX-DT, STARD4-AS1, PRC1-AS1, LINC03014, CACNA1C-AS1, MIR5193, NAV2-AS2, SGO1-AS1, THOC7-AS1, DIAPH3-AS1, APOBEC3B-AS1, TROAP-AS1, HMMR-AS1, LIPC-AS1, LINC02728, LINC01315, MIR1244-4, CZ1P-ASNS, KIF26B-AS1, GAPDH-DT, MIR155HG, A2M-AS1, MIR17HG, SERTAD4-AS1, HHIP-AS1, MIR924HG, SNORD86, PKD1L1-AS1, MIR503HG | Guide rRNA modifications and post-transcriptional control; shape immune and apoptotic responses. | Downregulated |
| Immune Response and Inflammation | SPAG5, CISH, FANCD2, FANCB, FANCG, IL17RD, FANCI, CXCL12, XRCC2, SOCS1, PRC1 | Cytokines, chemokines, antigen presentation; leukocyte recruitment and inflammatory signaling. | Downregulated |
| Signal Transduction and Cell Communication | NR2E3, CDK2, CDK4, CDK6, CDKN2C, CDKN3, ERN2, PLK4, CKS1B, CKS2, PAQR4, S1PR1, F2RL1, PASK, FLT1, LCORL, TRIB2, HUNK, HMMR, CERKL, LBR, MARCKS, NEK2, OXTR, PCK2, SCARA3, PDGFRA, PIM1, PLK1, HR, PBK, MAP2K6, ADGRG6, RGS7, RTP4, AURKA, BUB1, BUB1B, TK1, TRPC6 | Receptor/kinase/adapter circuits transmitting extracellular cues to transcriptional programs. | Downregulated |
| Metabolism and Cellular Homeostasis | TXNDC16 | Detoxification, redox balance, transporters and pumps; ion and nutrient homeostasis. | Downregulated |
| Structural and Cytoskeletal Components | TRAIP, ZWINT, OIP5, COL1A1, COL3A1, COL5A1, COL6A3, COL15A1, CKAP2L, ECM2, TPX2, MATCAP2, ASPM, RBMS3, PSMC3IP, MAP2, MMP11, COL5A3, CDON, PCOLCE, ANLN, PIMREG, COL14A1, BRIP1, DNAH11, TICRR, SNCAIP | Cytoskeleton and matrix components that support shape, transport and mechanics. | Downregulated |
| Neurotransmission and Nervous System Development | NLGN1, NPAS4, NNAT, NRGN, NETO2, FAXC, NREP | Synaptic transmission, ion channels, GPCRs and neuronal excitability. | Downregulated |
| Apoptosis and Cell Death | DDIAS, BCL2 | Effectors and regulators of programmed cell death and survival balance. | Downregulated |
| Transcription and Gene Regulation | DACH1, E2F1, E2F2, E2F3, EYA1, EYA2, EZH2, FOXM1, AUTS2, H1-3, H1-5, H2AX, H2AZ1, MAF, MN1, ASF1B, RBL1, SNAI1, SOX11, TCF19, E2F8, CDT1, CHAF1B, H2AC14, TCF7L1, H4C3, RUNX1T1 | Transcription factors and chromatin modulators governing gene expression programs. | Downregulated |
| DNA Replication and Repair | MMS22L, PCNA, MCM10, RFC2, RFC3, BRCA1, BRCA2, DSCC1, CDC45 | Replication origin licensing and DNA damage response/repair modules. | Downregulated |
| Cell Cycle Regulation and Proliferation | SKA1, SKA3, NUSAP1, GTSE1, ERCC6L, NCAPG2, SHCBP1, CCNF, PTTG1, ESPL1, CDC20 | Cyclins, CDKs and checkpoints that drive cell cycle transitions. | Downregulated |
| Miscellaneous Categories | ZNF730, CCDC152, TROAP, MAMLD1, SMC4, PHOSPHO2-KLHL23, ZNF670-ZNF695, GPC6, FSBP, TSPAN2, KIF20A, PLXNC1, SCML2, NDC80, SALRNA1, TACC3, RNASEH2A, FAM247A, FAM247D, FAM247C, CH507-145C22.1, CENPA, SMC2, CENPE, CENPF, POSTN, RAD51AP1, BUB1B-PAK6, PNMA2, CORIN, POLQ, DBF4, KIF2C, TMSB15A, RCC1, CNTRL, UBE2C, DEPP1, DMC1, LZTS1 | Stress responses, hormone/peptide signaling and other uncategorized functions. | Downregulated |

**Table 3. Senescence-related genes detected in acute and replicative comparisons with |log2FC| < 1, that fall below the primary effect-size threshold used for DEG calling.**

| **Category** | **Gene Symbols** | **Function** | **Dataset** |
| --- | --- | --- | --- |
| Cell Cycle Inhibitors | CDKN2B | p15INK4B; supports RB1-mediated growth arrest. | Acute |
| Cell Cycle Inhibitors | RB1 | Controls E2F; maintains G1 arrest. | Acute |
| Cell Cycle Inhibitors | TP53 | Tumor suppressor; activates p21 and checkpoints. | Acute |
| DNA Damage Response (DDR) | ATM | DSB sensor; activates TP53/H2AX. | Acute |
| DNA Damage Response (DDR) | ATR | Replication-stress checkpoint kinase. | Acute |
| DNA Damage Response (DDR) | CHEK1 | Effector kinase of ATR signaling. | Acute |
| DNA Damage Response (DDR) | CHEK2 | Effector kinase of ATM signaling. | Acute |
| Nuclear Envelope / Structural | LMNA | Nuclear structure; reorganized in senescence. | Acute |
| Nuclear Envelope / Structural | LMNB1 | Loss is a hallmark of senescence. | Acute |
| SASP Cytokines / Inflammation | TGFB1 | Fibrosis and feedback. | Acute |
| Matrix Remodeling | MMP14 | Membrane-type MMP. | Acute |
| Chromatin / Architectural | HMGA1 | Heterochromatin remodeling. | Acute |
| Chromatin / Architectural | HMGA2 | Chromatin architecture. | Acute |
| Cell Cycle Inhibitors | RB1 | Controls E2F; maintains G1 arrest. | Replicative |
| Cell Cycle Inhibitors | TP53 | Tumor suppressor; activates p21 and checkpoints. | Replicative |
| DNA Damage Response (DDR) | ATM | DSB sensor; activates TP53/H2AX. | Replicative |
| DNA Damage Response (DDR) | ATR | Replication-stress checkpoint kinase. | Replicative |
| DNA Damage Response (DDR) | CHEK2 | Effector kinase of ATM signaling. | Replicative |
| Nuclear Envelope / Structural | LMNA | Nuclear structure; reorganized in senescence. | Replicative |
| SASP Cytokines / Inflammation | CCL8 | Immune cell migration. | Replicative |
| Matrix Remodeling | MMP14 | Membrane-type MMP. | Replicative |
| Apoptosis / Death Receptors | TNFRSF10A | DR4; TRAIL apoptosis. | Replicative |
| Apoptosis / Death Receptors | TNFRSF10C | DcR1; decoy receptor. | Replicative |
| Apoptosis / Death Receptors | TNFSF9 | 4-1BB ligand; immune activation. | Replicative |
| Chromatin / Architectural | HMGA1 | Heterochromatin remodeling. | Replicative |
| Chromatin / Architectural | HMGA2 | Chromatin architecture. | Replicative |
| Chromatin / Architectural | HMGB1 | Alarmin; pro-inflammatory. | Replicative |
| Secreted / Growth Modulator | GDF15 | Stress-induced cytokine. | Replicative |

**Table 4. Commonly Upregulated GO Biological Process (GO:BP) Terms Shared Between Replicative and Acute Senescence**

| **ID** | **Description** | **Fisher_chisq** | **Fisher_padj** |
| --- | --- | --- | --- |
| GO:0050900 | leukocyte migration | 55.75064 | 2.26E-11 |
| GO:0006959 | humoral immune response | 43.50712 | 8.12E-09 |
| GO:0006816 | calcium ion transport | 31.08297 | 2.94E-06 |
| GO:0009612 | response to mechanical stimulus | 23.05104 | 1.24E-04 |
| GO:0098742 | cell-cell adhesion via plasma-membrane adhesion molecules | 76.24226 | 1.09E-15 |
| GO:0071621 | granulocyte chemotaxis | 45.32052 | 3.41E-09 |
| GO:0061844 | antimicrobial humoral immune response mediated by antimicrobial peptide | 26.37881 | 2.65E-05 |
| GO:0001906 | cell killing | 26.2057 | 2.88E-05 |
| GO:0044703 | multi-organism reproductive process | 19.85518 | 5.33E-04 |
| GO:0097530 | granulocyte migration | 44.26863 | 5.64E-09 |
| GO:0044706 | multi-multicellular organism process | 19.42846 | 6.47E-04 |
| GO:0043410 | positive regulation of MAPK cascade | 27.43084 | 1.63E-05 |
| GO:0055074 | calcium ion homeostasis | 30.77392 | 3.40E-06 |
| GO:0006935 | chemotaxis | 40.25461 | 3.83E-08 |
| GO:0042330 | taxis | 39.99825 | 4.33E-08 |
| GO:0006874 | intracellular calcium ion homeostasis | 28.13904 | 1.17E-05 |
| GO:0033627 | cell adhesion mediated by integrin | 18.80063 | 8.60E-04 |
| GO:0097529 | myeloid leukocyte migration | 49.90752 | 3.78E-10 |
| GO:0070371 | ERK1 and ERK2 cascade | 26.9123 | 2.07E-05 |
| GO:0030595 | leukocyte chemotaxis | 44.88762 | 4.20E-09 |
| GO:0043269 | regulation of monoatomic ion transport | 34.21489 | 6.73E-07 |
| GO:0031960 | response to corticosteroid | 18.45384 | 0.001006 |
| GO:0002274 | myeloid leukocyte activation | 22.88541 | 1.33E-04 |
| GO:0002429 | immune response-activating cell surface receptor signaling pathway | 18.0266 | 0.001219 |
| GO:0070372 | regulation of ERK1 and ERK2 cascade | 24.01781 | 7.92E-05 |
| GO:0019730 | antimicrobial humoral response | 33.30877 | 1.03E-06 |
| GO:0072676 | lymphocyte migration | 23.66458 | 9.32E-05 |
| GO:1902644 | tertiary alcohol metabolic process | 18.14049 | 0.001158 |
| GO:0051384 | response to glucocorticoid | 19.22916 | 7.09E-04 |
| GO:0001894 | tissue homeostasis | 20.26529 | 4.43E-04 |
| GO:0060249 | anatomical structure homeostasis | 20.26529 | 4.43E-04 |
| GO:0043491 | phosphatidylinositol 3-kinase/protein kinase B signal transduction | 18.62136 | 9.33E-04 |
| GO:0002703 | regulation of leukocyte mediated immunity | 18.06165 | 0.0012 |
| GO:0070588 | calcium ion transmembrane transport | 22.6317 | 1.50E-04 |
| GO:0021700 | developmental maturation | 19.94438 | 5.12E-04 |
| GO:0002685 | regulation of leukocyte migration | 35.55941 | 3.57E-07 |
| GO:0042391 | regulation of membrane potential | 49.8596 | 3.86E-10 |
| GO:0060326 | cell chemotaxis | 37.14309 | 1.68E-07 |
| GO:0006814 | sodium ion transport | 27.3788 | 1.67E-05 |
| GO:0002768 | immune response-regulating cell surface receptor signaling pathway | 19.15999 | 7.31E-04 |
| GO:0150077 | regulation of neuroinflammatory response | 20.13651 | 4.69E-04 |
| GO:0031640 | killing of cells of another organism | 17.83473 | 0.001329 |
| GO:0141061 | disruption of cell in another organism | 17.83473 | 0.001329 |
| GO:1990266 | neutrophil migration | 25.19344 | 4.60E-05 |
| GO:0030593 | neutrophil chemotaxis | 24.40387 | 6.63E-05 |
| GO:0070663 | regulation of leukocyte proliferation | 19.99325 | 5.01E-04 |
| GO:0072593 | reactive oxygen species metabolic process | 24.67618 | 5.84E-05 |
| GO:0141060 | disruption of anatomical structure in another organism | 16.79173 | 0.002122 |
| GO:0035725 | sodium ion transmembrane transport | 21.91789 | 2.08E-04 |
| GO:0120254 | olefinic compound metabolic process | 29.5876 | 5.94E-06 |
| GO:0016101 | diterpenoid metabolic process | 24.46757 | 6.44E-05 |
| GO:0070661 | leukocyte proliferation | 18.23022 | 0.001113 |
| GO:0034308 | primary alcohol metabolic process | 22.9461 | 1.30E-04 |
| GO:0070665 | positive regulation of leukocyte proliferation | 16.08935 | 0.002902 |
| GO:0006836 | neurotransmitter transport | 15.24264 | 0.004224 |
| GO:1990868 | response to chemokine | 27.80728 | 1.36E-05 |
| GO:1990869 | cellular response to chemokine | 27.80728 | 1.36E-05 |
| GO:0048245 | eosinophil chemotaxis | 25.96048 | 3.22E-05 |
| GO:0071674 | mononuclear cell migration | 34.73446 | 5.27E-07 |
| GO:0070098 | chemokine-mediated signaling pathway | 27.58646 | 1.51E-05 |
| GO:0050920 | regulation of chemotaxis | 29.62666 | 5.83E-06 |
| GO:1900745 | positive regulation of p38MAPK cascade | 14.73798 | 0.005277 |
| GO:0001523 | retinoid metabolic process | 24.768 | 5.60E-05 |
| GO:0051896 | regulation of phosphatidylinositol 3-kinase/protein kinase B signal transduction | 16.93068 | 0.001994 |
| GO:0043062 | extracellular structure organization | 30.90593 | 3.20E-06 |
| GO:0015711 | organic anion transport | 14.23305 | 0.006587 |
| GO:0051897 | positive regulation of phosphatidylinositol 3-kinase/protein kinase B signal transduction | 16.52142 | 0.002394 |
| GO:0038128 | ERBB2 signaling pathway | 17.23142 | 0.001743 |
| GO:0051924 | regulation of calcium ion transport | 17.73074 | 0.001393 |
| GO:0006721 | terpenoid metabolic process | 22.31729 | 1.73E-04 |
| GO:0042116 | macrophage activation | 17.67644 | 0.001427 |
| GO:0002548 | monocyte chemotaxis | 21.13975 | 2.97E-04 |
| GO:0070374 | positive regulation of ERK1 and ERK2 cascade | 18.77329 | 8.71E-04 |
| GO:0030638 | polyketide metabolic process | 14.074 | 0.007063 |
| GO:0030647 | aminoglycoside antibiotic metabolic process | 14.074 | 0.007063 |
| GO:0044598 | doxorubicin metabolic process | 14.074 | 0.007063 |
| GO:2000425 | regulation of apoptotic cell clearance | 14.074 | 0.007063 |
| GO:0050670 | regulation of lymphocyte proliferation | 16.23013 | 0.002725 |
| GO:0072677 | eosinophil migration | 21.99542 | 2.01E-04 |
| GO:0010959 | regulation of metal ion transport | 24.78808 | 5.55E-05 |
| GO:0009410 | response to xenobiotic stimulus | 15.87514 | 0.003191 |
| GO:0030198 | extracellular matrix organization | 30.17174 | 4.52E-06 |
| GO:0071695 | anatomical structure maturation | 19.75852 | 5.57E-04 |
| GO:0045229 | external encapsulating structure organization | 29.95539 | 5.00E-06 |
| GO:0032944 | regulation of mononuclear cell proliferation | 15.46757 | 0.003824 |
| GO:0006911 | phagocytosis, engulfment | 14.09565 | 0.006996 |
| GO:0006936 | muscle contraction | 20.6258 | 3.76E-04 |
| GO:0071492 | cellular response to UV-A | 16.40919 | 0.002516 |
| GO:0097553 | calcium ion transmembrane import into cytosol | 17.83937 | 0.001327 |
| GO:0008544 | epidermis development | 14.39636 | 0.006132 |
| GO:0061900 | glial cell activation | 18.97742 | 7.94E-04 |
| GO:0002281 | macrophage activation involved in immune response | 14.94688 | 0.004813 |
| GO:0003012 | muscle system process | 24.75301 | 5.64E-05 |
| GO:0042445 | hormone metabolic process | 34.31936 | 6.41E-07 |
| GO:1901654 | response to ketone | 12.97122 | 0.011417 |
| GO:0002687 | positive regulation of leukocyte migration | 27.94879 | 1.28E-05 |
| GO:0002275 | myeloid cell activation involved in immune response | 15.95476 | 0.00308 |
| GO:0001774 | microglial cell activation | 18.9887 | 7.90E-04 |
| GO:0007156 | homophilic cell adhesion via plasma membrane adhesion molecules | 68.30021 | 5.18E-14 |
| GO:1903409 | reactive oxygen species biosynthetic process | 13.79424 | 0.007982 |
| GO:0002688 | regulation of leukocyte chemotaxis | 25.17415 | 4.64E-05 |
| GO:0150076 | neuroinflammatory response | 22.06016 | 1.95E-04 |
| GO:2000379 | positive regulation of reactive oxygen species metabolic process | 28.47303 | 1.00E-05 |
| GO:0046942 | carboxylic acid transport | 13.52014 | 0.008995 |
| GO:0015849 | organic acid transport | 13.40795 | 0.009445 |
| GO:0042572 | retinol metabolic process | 15.72551 | 0.00341 |
| GO:0002269 | leukocyte activation involved in inflammatory response | 17.6069 | 0.001473 |
| GO:0022617 | extracellular matrix disassembly | 23.68701 | 9.23E-05 |
| GO:0046651 | lymphocyte proliferation | 13.07288 | 0.010925 |
| GO:0050921 | positive regulation of chemotaxis | 28.78512 | 8.64E-06 |
| GO:0050806 | positive regulation of synaptic transmission | 12.22818 | 0.015733 |
| GO:0070141 | response to UV-A | 13.46586 | 0.00921 |

**Table 5. Commonly Downregulated GO Biological Process (GO:BP) Terms Shared Between Replicative and Acute Senescence**

| **ID** | **Description** | **Fisher_chisq** | **Fisher_padj** |
| --- | --- | --- | --- |
| GO:0007059 | chromosome segregation | 456.9069 | 1.40E-97 |
| GO:0000280 | nuclear division | 441.0534 | 3.73E-94 |
| GO:0098813 | nuclear chromosome segregation | 403.9759 | 3.85E-86 |
| GO:0048285 | organelle fission | 417.5925 | 4.39E-89 |
| GO:0140014 | mitotic nuclear division | 404.7448 | 2.62E-86 |
| GO:0000070 | mitotic sister chromatid segregation | 371.7877 | 3.46E-79 |
| GO:0000819 | sister chromatid segregation | 363.6893 | 1.94E-77 |
| GO:0044772 | mitotic cell cycle phase transition | 328.8511 | 6.45E-70 |
| GO:1901987 | regulation of cell cycle phase transition | 290.2776 | 1.35E-61 |
| GO:0006260 | DNA replication | 311.3438 | 3.87E-66 |
| GO:0006261 | DNA-templated DNA replication | 278.9409 | 3.77E-59 |
| GO:1901990 | regulation of mitotic cell cycle phase transition | 261.5864 | 2.08E-55 |
| GO:0090068 | positive regulation of cell cycle process | 244.0908 | 1.22E-51 |
| GO:0045787 | positive regulation of cell cycle | 228.8648 | 2.32E-48 |
| GO:0051983 | regulation of chromosome segregation | 247.8714 | 1.87E-52 |
| GO:0007088 | regulation of mitotic nuclear division | 258.6009 | 9.13E-55 |
| GO:1905818 | regulation of chromosome separation | 234.0177 | 1.80E-49 |
| GO:0010948 | negative regulation of cell cycle process | 229.2507 | 1.91E-48 |
| GO:0051783 | regulation of nuclear division | 235.2998 | 9.54E-50 |
| GO:0051304 | chromosome separation | 221.2895 | 9.90E-47 |
| GO:1902850 | microtubule cytoskeleton organization involved in mitosis | 200.9612 | 2.33E-42 |
| GO:0000075 | cell cycle checkpoint signaling | 214.4062 | 3.00E-45 |
| GO:0051321 | meiotic cell cycle | 191.3531 | 2.71E-40 |
| GO:0007051 | spindle organization | 191.2318 | 2.88E-40 |
| GO:1901988 | negative regulation of cell cycle phase transition | 201.9597 | 1.42E-42 |
| GO:0045786 | negative regulation of cell cycle | 205.877 | 2.05E-43 |
| GO:0051310 | metaphase chromosome alignment | 203.1501 | 7.90E-43 |
| GO:1903046 | meiotic cell cycle process | 184.6953 | 7.31E-39 |
| GO:0007052 | mitotic spindle organization | 178.1029 | 1.91E-37 |
| GO:0044839 | cell cycle G2/M phase transition | 161.0361 | 8.76E-34 |
| GO:0010965 | regulation of mitotic sister chromatid separation | 195.9937 | 2.73E-41 |
| GO:0050000 | chromosome localization | 189.1512 | 8.07E-40 |
| GO:0044786 | cell cycle DNA replication | 177.6064 | 2.44E-37 |
| GO:0051306 | mitotic sister chromatid separation | 190.6203 | 3.90E-40 |
| GO:0051784 | negative regulation of nuclear division | 190.6203 | 3.90E-40 |
| GO:0051303 | establishment of chromosome localization | 188.5362 | 1.09E-39 |
| GO:0045839 | negative regulation of mitotic nuclear division | 190.0303 | 5.22E-40 |
| GO:0033045 | regulation of sister chromatid segregation | 187.004 | 2.33E-39 |
| GO:0140013 | meiotic nuclear division | 164.9403 | 1.27E-34 |
| GO:0000086 | G2/M transition of mitotic cell cycle | 149.3596 | 2.79E-31 |
| GO:1901991 | negative regulation of mitotic cell cycle phase transition | 175.9405 | 5.55E-37 |
| GO:0007091 | metaphase/anaphase transition of mitotic cell cycle | 180.4612 | 5.94E-38 |
| GO:0033044 | regulation of chromosome organization | 164.4494 | 1.62E-34 |
| GO:0033046 | negative regulation of sister chromatid segregation | 182.5861 | 2.08E-38 |
| GO:0033048 | negative regulation of mitotic sister chromatid segregation | 182.5861 | 2.08E-38 |
| GO:0045841 | negative regulation of mitotic metaphase/anaphase transition | 182.5861 | 2.08E-38 |
| GO:2000816 | negative regulation of mitotic sister chromatid separation | 182.5861 | 2.08E-38 |
| GO:0007093 | mitotic cell cycle checkpoint signaling | 179.7431 | 8.47E-38 |
| GO:0033047 | regulation of mitotic sister chromatid segregation | 185.961 | 3.91E-39 |
| GO:0044784 | metaphase/anaphase transition of cell cycle | 177.1562 | 3.04E-37 |
| GO:1902100 | negative regulation of metaphase/anaphase transition of cell cycle | 178.4154 | 1.63E-37 |
| GO:1905819 | negative regulation of chromosome separation | 178.4154 | 1.63E-37 |
| GO:0033260 | nuclear DNA replication | 161.1098 | 8.45E-34 |
| GO:0030071 | regulation of mitotic metaphase/anaphase transition | 175.8943 | 5.68E-37 |
| GO:0051985 | negative regulation of chromosome segregation | 182.178 | 2.54E-38 |
| GO:0045930 | negative regulation of mitotic cell cycle | 163.1163 | 3.14E-34 |
| GO:1902099 | regulation of metaphase/anaphase transition of cell cycle | 172.161 | 3.59E-36 |
| GO:0007094 | mitotic spindle assembly checkpoint signaling | 174.6669 | 1.04E-36 |
| GO:0071173 | spindle assembly checkpoint signaling | 174.6669 | 1.04E-36 |
| GO:0071174 | mitotic spindle checkpoint signaling | 174.6669 | 1.04E-36 |
| GO:1901989 | positive regulation of cell cycle phase transition | 138.2041 | 6.84E-29 |
| GO:0006310 | DNA recombination | 146.8512 | 9.62E-31 |
| GO:0031577 | spindle checkpoint signaling | 172.6145 | 2.87E-36 |
| GO:0010389 | regulation of G2/M transition of mitotic cell cycle | 139.7611 | 3.18E-29 |
| GO:1902749 | regulation of cell cycle G2/M phase transition | 136.7996 | 1.37E-28 |
| GO:0008608 | attachment of spindle microtubules to kinetochore | 164.428 | 1.64E-34 |
| GO:0006302 | double-strand break repair | 149.6762 | 2.39E-31 |
| GO:0045931 | positive regulation of mitotic cell cycle | 129.917 | 4.06E-27 |
| GO:1901992 | positive regulation of mitotic cell cycle phase transition | 126.3264 | 2.38E-26 |
| GO:0006270 | DNA replication initiation | 148.2032 | 4.94E-31 |
| GO:0045132 | meiotic chromosome segregation | 128.7843 | 7.09E-27 |
| GO:0090329 | regulation of DNA-templated DNA replication | 129.2349 | 5.68E-27 |
| GO:0051225 | spindle assembly | 127.9133 | 1.09E-26 |
| GO:0061982 | meiosis I cell cycle process | 124.7993 | 5.04E-26 |
| GO:0006275 | regulation of DNA replication | 122.9245 | 1.27E-25 |
| GO:0051984 | positive regulation of chromosome segregation | 114.7006 | 7.23E-24 |
| GO:0000725 | recombinational repair | 130.104 | 3.70E-27 |
| GO:2001251 | negative regulation of chromosome organization | 125.2168 | 4.10E-26 |
| GO:1905820 | positive regulation of chromosome separation | 116.1466 | 3.55E-24 |
| GO:0140694 | membraneless organelle assembly | 103.107 | 2.14E-21 |
| GO:0000724 | double-strand break repair via homologous recombination | 129.9916 | 3.91E-27 |
| GO:0000727 | double-strand break repair via break-induced replication | 108.0802 | 1.87E-22 |
| GO:1902751 | positive regulation of cell cycle G2/M phase transition | 83.38897 | 3.33E-17 |
| GO:0007080 | mitotic metaphase chromosome alignment | 115.23 | 5.57E-24 |
| GO:0007127 | meiosis I | 99.24211 | 1.43E-20 |
| GO:1902969 | mitotic DNA replication | 109.5377 | 9.13E-23 |
| GO:0010971 | positive regulation of G2/M transition of mitotic cell cycle | 76.93357 | 7.77E-16 |
| GO:0051656 | establishment of organelle localization | 105.8222 | 5.66E-22 |
| GO:0090307 | mitotic spindle assembly | 92.32401 | 4.22E-19 |
| GO:1901976 | regulation of cell cycle checkpoint | 92.05807 | 4.81E-19 |
| GO:0051383 | kinetochore organization | 89.30812 | 1.85E-18 |
| GO:0044771 | meiotic cell cycle phase transition | 79.72349 | 1.99E-16 |
| GO:0044843 | cell cycle G1/S phase transition | 85.31928 | 1.30E-17 |
| GO:0000082 | G1/S transition of mitotic cell cycle | 82.79764 | 4.45E-17 |
| GO:0034508 | centromere complex assembly | 94.17263 | 1.71E-19 |
| GO:0000910 | cytokinesis | 74.98168 | 2.01E-15 |
| GO:0031570 | DNA integrity checkpoint signaling | 83.91035 | 2.58E-17 |
| GO:0030261 | chromosome condensation | 70.22706 | 2.03E-14 |
| GO:0051231 | spindle elongation | 89.21783 | 1.93E-18 |
| GO:0045005 | DNA-templated DNA replication maintenance of fidelity | 80.22886 | 1.56E-16 |
| GO:0051315 | attachment of mitotic spindle microtubules to kinetochore | 90.03156 | 1.30E-18 |
| GO:0051256 | mitotic spindle midzone assembly | 85.82446 | 1.01E-17 |
| GO:0030174 | regulation of DNA-templated DNA replication initiation | 82.08801 | 6.29E-17 |
| GO:0000281 | mitotic cytokinesis | 69.78929 | 2.51E-14 |
| GO:0010639 | negative regulation of organelle organization | 71.35551 | 1.17E-14 |
| GO:0000022 | mitotic spindle elongation | 81.2123 | 9.64E-17 |
| GO:0051988 | regulation of attachment of spindle microtubules to kinetochore | 90.52924 | 1.02E-18 |
| GO:0090231 | regulation of spindle checkpoint | 73.37584 | 4.39E-15 |
| GO:0007143 | female meiotic nuclear division | 61.62858 | 1.32E-12 |
| GO:0007062 | sister chromatid cohesion | 63.75775 | 4.70E-13 |
| GO:0051054 | positive regulation of DNA metabolic process | 63.69327 | 4.85E-13 |
| GO:0051255 | spindle midzone assembly | 73.67792 | 3.79E-15 |
| GO:0065004 | protein-DNA complex assembly | 83.95178 | 2.53E-17 |
| GO:0034502 | protein localization to chromosome | 78.20693 | 4.18E-16 |
| GO:0006271 | DNA strand elongation involved in DNA replication | 77.8906 | 4.87E-16 |
| GO:1901970 | positive regulation of mitotic sister chromatid separation | 69.03016 | 3.64E-14 |
| GO:0032465 | regulation of cytokinesis | 63.30146 | 5.86E-13 |
| GO:0061640 | cytoskeleton-dependent cytokinesis | 53.99484 | 5.28E-11 |
| GO:0071824 | protein-DNA complex organization | 75.99361 | 1.23E-15 |
| GO:0090266 | regulation of mitotic cell cycle spindle assembly checkpoint | 63.10323 | 6.45E-13 |
| GO:1903504 | regulation of mitotic spindle checkpoint | 63.10323 | 6.45E-13 |
| GO:0071459 | protein localization to chromosome, centromeric region | 75.92239 | 1.27E-15 |
| GO:0007076 | mitotic chromosome condensation | 49.13599 | 5.47E-10 |
| GO:0035825 | homologous recombination | 55.11833 | 3.07E-11 |
| GO:0051445 | regulation of meiotic cell cycle | 47.2669 | 1.34E-09 |
| GO:0051302 | regulation of cell division | 56.85737 | 1.33E-11 |
| GO:1901993 | regulation of meiotic cell cycle phase transition | 46.60697 | 1.84E-09 |
| GO:0007098 | centrosome cycle | 58.0312 | 7.52E-12 |
| GO:0051338 | regulation of transferase activity | 43.65206 | 7.58E-09 |
| GO:0000079 | regulation of cyclin-dependent protein serine/threonine kinase activity | 48.3725 | 7.89E-10 |
| GO:0000077 | DNA damage checkpoint signaling | 52.48727 | 1.09E-10 |
| GO:0034501 | protein localization to kinetochore | 56.61455 | 1.49E-11 |
| GO:0090306 | meiotic spindle assembly | 50.31348 | 3.11E-10 |
| GO:1903083 | protein localization to condensed chromosome | 56.61455 | 1.49E-11 |
| GO:0000212 | meiotic spindle organization | 49.47935 | 4.64E-10 |
| GO:0031297 | replication fork processing | 47.04785 | 1.49E-09 |
| GO:0031055 | chromatin remodeling at centromere | 65.26108 | 2.27E-13 |
| GO:0034080 | CENP-A containing chromatin assembly | 65.26108 | 2.27E-13 |
| GO:2001252 | positive regulation of chromosome organization | 44.23886 | 5.72E-09 |
| GO:0042770 | signal transduction in response to DNA damage | 49.66383 | 4.24E-10 |
| GO:0070192 | chromosome organization involved in meiotic cell cycle | 41.65441 | 1.97E-08 |
| GO:0031023 | microtubule organizing center organization | 52.41094 | 1.13E-10 |
| GO:0009314 | response to radiation | 38.44631 | 9.06E-08 |
| GO:0045143 | homologous chromosome segregation | 43.72505 | 7.32E-09 |
| GO:0051382 | kinetochore assembly | 52.18479 | 1.26E-10 |
| GO:0045740 | positive regulation of DNA replication | 44.34335 | 5.44E-09 |
| GO:0007056 | spindle assembly involved in female meiosis | 44.15803 | 5.95E-09 |
| GO:0000731 | DNA synthesis involved in DNA repair | 49.90907 | 3.77E-10 |
| GO:0022616 | DNA strand elongation | 45.94599 | 2.53E-09 |
| GO:0010972 | negative regulation of G2/M transition of mitotic cell cycle | 46.86796 | 1.62E-09 |
| GO:0006282 | regulation of DNA repair | 37.97227 | 1.14E-07 |
| GO:1902750 | negative regulation of cell cycle G2/M phase transition | 45.65742 | 2.90E-09 |
| GO:0007292 | female gamete generation | 43.62669 | 7.67E-09 |
| GO:0000076 | DNA replication checkpoint signaling | 46.72753 | 1.74E-09 |
| GO:0051447 | negative regulation of meiotic cell cycle | 41.46958 | 2.15E-08 |
| GO:0007144 | female meiosis I | 36.48135 | 2.30E-07 |
| GO:0060623 | regulation of chromosome condensation | 36.48135 | 2.30E-07 |
| GO:0090232 | positive regulation of spindle checkpoint | 48.23884 | 8.42E-10 |
| GO:0071897 | DNA biosynthetic process | 56.70306 | 1.43E-11 |
| GO:2000105 | positive regulation of DNA-templated DNA replication | 34.71849 | 5.31E-07 |
| GO:0048144 | fibroblast proliferation | 41.3501 | 2.27E-08 |
| GO:0051653 | spindle localization | 36.98414 | 1.81E-07 |
| GO:0044818 | mitotic G2/M transition checkpoint | 43.49945 | 8.15E-09 |
| GO:0032886 | regulation of microtubule-based process | 40.22048 | 3.90E-08 |
| GO:0007131 | reciprocal meiotic recombination | 37.07607 | 1.74E-07 |
| GO:0140527 | reciprocal homologous recombination | 37.07607 | 1.74E-07 |
| GO:0045835 | negative regulation of meiotic nuclear division | 35.7787 | 3.21E-07 |
| GO:0051307 | meiotic chromosome separation | 35.7787 | 3.21E-07 |
| GO:0009411 | response to UV | 26.84793 | 2.13E-05 |
| GO:0044774 | mitotic DNA integrity checkpoint signaling | 39.00058 | 6.96E-08 |
| GO:0045842 | positive regulation of mitotic metaphase/anaphase transition | 34.95228 | 4.75E-07 |
| GO:0090224 | regulation of spindle organization | 33.12844 | 1.12E-06 |
| GO:0051293 | establishment of spindle localization | 32.66776 | 1.40E-06 |
| GO:0019985 | translesion synthesis | 34.09403 | 7.13E-07 |
| GO:0006301 | postreplication repair | 33.05553 | 1.16E-06 |
| GO:0010212 | response to ionizing radiation | 32.18614 | 1.75E-06 |
| GO:1902101 | positive regulation of metaphase/anaphase transition of cell cycle | 33.57115 | 9.12E-07 |
| GO:0051987 | positive regulation of attachment of spindle microtubules to kinetochore | 33.65634 | 8.76E-07 |
| GO:0032467 | positive regulation of cytokinesis | 34.59682 | 5.62E-07 |
| GO:0070507 | regulation of microtubule cytoskeleton organization | 32.57316 | 1.46E-06 |
| GO:1901978 | positive regulation of cell cycle checkpoint | 37.24442 | 1.60E-07 |
| GO:0090267 | positive regulation of mitotic cell cycle spindle assembly checkpoint | 37.54877 | 1.39E-07 |
| GO:0036297 | interstrand cross-link repair | 32.95305 | 1.22E-06 |
| GO:0007064 | mitotic sister chromatid cohesion | 29.44242 | 6.36E-06 |
| GO:0051053 | negative regulation of DNA metabolic process | 30.77903 | 3.40E-06 |
| GO:0060236 | regulation of mitotic spindle organization | 28.08403 | 1.20E-05 |
| GO:0000018 | regulation of DNA recombination | 29.50896 | 6.16E-06 |
| GO:0040020 | regulation of meiotic nuclear division | 27.83329 | 1.35E-05 |
| GO:0006287 | base-excision repair, gap-filling | 28.51313 | 9.82E-06 |
| GO:2000241 | regulation of reproductive process | 25.14832 | 4.70E-05 |
| GO:0071478 | cellular response to radiation | 23.51562 | 9.99E-05 |
| GO:0045840 | positive regulation of mitotic nuclear division | 29.58707 | 5.94E-06 |
| GO:0051294 | establishment of spindle orientation | 26.27128 | 2.79E-05 |
| GO:0051785 | positive regulation of nuclear division | 28.29399 | 1.09E-05 |
| GO:0044849 | estrous cycle | 22.54061 | 1.56E-04 |
| GO:0090235 | regulation of metaphase plate congression | 37.90084 | 1.17E-07 |
| GO:0007095 | mitotic G2 DNA damage checkpoint signaling | 28.57018 | 9.56E-06 |
| GO:0051781 | positive regulation of cell division | 22.61124 | 1.51E-04 |
| GO:0045910 | negative regulation of DNA recombination | 23.46026 | 1.02E-04 |
| GO:0007063 | regulation of sister chromatid cohesion | 20.37428 | 4.21E-04 |
| GO:0048146 | positive regulation of fibroblast proliferation | 25.5642 | 3.87E-05 |
| GO:0030010 | establishment of cell polarity | 26.07801 | 3.05E-05 |
| GO:0031109 | microtubule polymerization or depolymerization | 20.39175 | 4.18E-04 |
| GO:0044773 | mitotic DNA damage checkpoint signaling | 27.45784 | 1.61E-05 |
| GO:1902806 | regulation of cell cycle G1/S phase transition | 24.67745 | 5.84E-05 |
| GO:0033313 | meiotic cell cycle checkpoint signaling | 29.24928 | 6.96E-06 |
| GO:1902423 | regulation of attachment of mitotic spindle microtubules to kinetochore | 35.77803 | 3.21E-07 |
| GO:2000104 | negative regulation of DNA-templated DNA replication | 29.24928 | 6.96E-06 |
| GO:0040001 | establishment of mitotic spindle localization | 21.89802 | 2.10E-04 |
| GO:1904666 | regulation of ubiquitin protein ligase activity | 26.93006 | 2.05E-05 |
| GO:0140588 | chromatin looping | 31.43654 | 2.49E-06 |
| GO:0042698 | ovulation cycle | 22.47318 | 1.61E-04 |
| GO:0071479 | cellular response to ionizing radiation | 22.47318 | 1.61E-04 |
| GO:0033314 | mitotic DNA replication checkpoint signaling | 27.41272 | 1.64E-05 |
| GO:0072711 | cellular response to hydroxyurea | 22.00456 | 2.00E-04 |
| GO:1990918 | double-strand break repair involved in meiotic recombination | 22.00456 | 2.00E-04 |
| GO:0051298 | centrosome duplication | 22.10224 | 1.91E-04 |
| GO:2000242 | negative regulation of reproductive process | 19.58012 | 6.04E-04 |
| GO:0006284 | base-excision repair | 26.96181 | 2.02E-05 |
| GO:0051347 | positive regulation of transferase activity | 19.55909 | 6.10E-04 |
| GO:2000045 | regulation of G1/S transition of mitotic cell cycle | 20.66794 | 3.68E-04 |
| GO:0071168 | protein localization to chromatin | 31.45029 | 2.48E-06 |
| GO:0072710 | response to hydroxyurea | 20.62819 | 0.000375 |
| GO:0045739 | positive regulation of DNA repair | 18.7841 | 8.67E-04 |
| GO:0010569 | regulation of double-strand break repair via homologous recombination | 22.57225 | 1.54E-04 |
| GO:0001556 | oocyte maturation | 18.72842 | 8.89E-04 |
| GO:2000001 | regulation of DNA damage checkpoint | 18.94579 | 8.05E-04 |
| GO:0000723 | telomere maintenance | 17.71606 | 0.001402 |
| GO:1903490 | positive regulation of mitotic cytokinesis | 24.352 | 6.79E-05 |
| GO:0007019 | microtubule depolymerization | 17.54783 | 0.001512 |
| GO:0008156 | negative regulation of DNA replication | 22.18712 | 1.84E-04 |
| GO:2000779 | regulation of double-strand break repair | 23.43682 | 1.04E-04 |
| GO:0032506 | cytokinetic process | 17.12681 | 0.001826 |
| GO:0040029 | epigenetic regulation of gene expression | 25.4512 | 4.08E-05 |
| GO:0032200 | telomere organization | 28.18249 | 1.15E-05 |
| GO:0007129 | homologous chromosome pairing at meiosis | 16.75937 | 0.002152 |
| GO:2000573 | positive regulation of DNA biosynthetic process | 22.6382 | 1.50E-04 |
| GO:0003180 | aortic valve morphogenesis | 16.6844 | 0.002226 |
| GO:1902808 | positive regulation of cell cycle G1/S phase transition | 21.00074 | 3.17E-04 |
| GO:2000042 | negative regulation of double-strand break repair via homologous recombination | 16.93631 | 0.001989 |
| GO:0048145 | regulation of fibroblast proliferation | 17.39052 | 0.001623 |
| GO:0001833 | inner cell mass cell proliferation | 21.89502 | 2.10E-04 |
| GO:0051299 | centrosome separation | 20.80205 | 3.47E-04 |
| GO:1902412 | regulation of mitotic cytokinesis | 20.80205 | 3.47E-04 |
| GO:0031099 | regeneration | 18.8444 | 8.43E-04 |
| GO:0007163 | establishment or maintenance of cell polarity | 17.98812 | 0.001241 |
| GO:0051782 | negative regulation of cell division | 24.43815 | 6.52E-05 |
| GO:0061351 | neural precursor cell proliferation | 15.90069 | 0.003155 |
| GO:0045814 | negative regulation of gene expression, epigenetic | 22.17066 | 1.85E-04 |
| GO:0003176 | aortic valve development | 16.7417 | 0.00217 |
| GO:0010458 | exit from mitosis | 19.66486 | 5.82E-04 |
| GO:1905314 | semi-lunar valve development | 15.03963 | 0.00462 |
| GO:0001701 | in utero embryonic development | 15.007 | 0.004687 |
| GO:0007096 | regulation of exit from mitosis | 16.96776 | 0.001961 |
| GO:0006298 | mismatch repair | 15.05607 | 0.004586 |
| GO:0051438 | regulation of ubiquitin-protein transferase activity | 14.58477 | 0.005645 |
| GO:0001832 | blastocyst growth | 14.80772 | 0.005117 |
| GO:2000278 | regulation of DNA biosynthetic process | 15.98911 | 0.003034 |
| GO:0031145 | anaphase-promoting complex-dependent catabolic process | 14.20299 | 0.006675 |
| GO:0006334 | nucleosome assembly | 26.37907 | 2.65E-05 |
| GO:0006312 | mitotic recombination | 13.09351 | 0.010828 |
| GO:0003179 | heart valve morphogenesis | 13.11885 | 0.010709 |
| GO:0007100 | mitotic centrosome separation | 13.61056 | 0.008648 |

**Table 6. Reactome pathways upregulated in both acute and replicative senescence models.**

| **ID** | **Description** | **Fisher_chisq** | **Fisher_padj** |
| --- | --- | --- | --- |
| R-HSA-6783783 | Interleukin-10 signaling | 33.21947 | 1.08E-06 |
| R-HSA-1474244 | Extracellular matrix organization | 34.23668 | 6.66E-07 |
| R-HSA-380108 | Chemokine receptors bind chemokines | 41.21789 | 2.42E-08 |
| R-HSA-1474228 | Degradation of the extracellular matrix | 23.97192 | 8.09E-05 |
| R-HSA-6785807 | Interleukin-4 and Interleukin-13 signaling | 25.65652 | 3.71E-05 |
| R-HSA-500792 | GPCR ligand binding | 20.16538 | 4.63E-04 |
| R-HSA-373076 | Class A/1 (Rhodopsin-like receptors) | 24.50701 | 6.32E-05 |
| R-HSA-202733 | Cell surface interactions at the vascular wall | 24.59541 | 6.07E-05 |
| R-HSA-375276 | Peptide ligand-binding receptors | 25.395 | 4.19E-05 |
| R-HSA-983712 | Ion channel transport | 18.6423 | 9.24E-04 |
| R-HSA-1369062 | ABC transporters in lipid homeostasis | 16.8647 | 0.002054 |
| R-HSA-381426 | Regulation of Insulin-like Growth Factor (IGF) transport and uptake by Insulin-like Growth Factor Binding Proteins (IGFBPs) | 16.10764 | 0.002878 |
| R-HSA-2672351 | Stimuli-sensing channels | 12.72668 | 0.012691 |

**Table 7. Reactome pathways downregulated in both acute and replicative senescence models.**

| **ID** | **Description** | **Fisher_chisq** | **Fisher_padj** |
| --- | --- | --- | --- |
| R-HSA-69620 | Cell Cycle Checkpoints | 369.786 | 9.36E-79 |
| R-HSA-453279 | Mitotic G1 phase and G1/S transition | 230.4801 | 1.04E-48 |
| R-HSA-68877 | Mitotic Prometaphase | 205.927 | 2.00E-43 |
| R-HSA-2500257 | Resolution of Sister Chromatid Cohesion | 202.6589 | 1.01E-42 |
| R-HSA-141424 | Amplification of signal from the kinetochores | 200.3919 | 3.09E-42 |
| R-HSA-141444 | Amplification of signal from unattached kinetochores via a MAD2 inhibitory signal | 200.3919 | 3.09E-42 |
| R-HSA-68886 | M Phase | 215.4973 | 1.74E-45 |
| R-HSA-69618 | Mitotic Spindle Checkpoint | 194.5949 | 5.46E-41 |
| R-HSA-69206 | G1/S Transition | 196.7344 | 1.89E-41 |
| R-HSA-2555396 | Mitotic Metaphase and Anaphase | 194.0842 | 7.02E-41 |
| R-HSA-68882 | Mitotic Anaphase | 186.7946 | 2.59E-39 |
| R-HSA-69242 | S Phase | 190.4402 | 4.26E-40 |
| R-HSA-9648025 | EML4 and NUDC in mitotic spindle formation | 179.4464 | 9.80E-38 |
| R-HSA-68962 | Activation of the pre-replicative complex | 182.191 | 2.52E-38 |
| R-HSA-5663220 | RHO GTPases Activate Formins | 164.3325 | 1.72E-34 |
| R-HSA-2467813 | Separation of Sister Chromatids | 161.6201 | 6.57E-34 |
| R-HSA-69481 | G2/M Checkpoints | 166.7692 | 5.16E-35 |
| R-HSA-176187 | Activation of ATR in response to replication stress | 176.7961 | 3.64E-37 |
| R-HSA-69190 | DNA strand elongation | 178.0844 | 1.92E-37 |
| R-HSA-69239 | Synthesis of DNA | 167.0235 | 4.55E-35 |
| R-HSA-69306 | DNA Replication | 179.4282 | 9.89E-38 |
| R-HSA-73886 | Chromosome Maintenance | 146.5867 | 1.10E-30 |
| R-HSA-69273 | Cyclin A/B1/B2 associated events during G2/M transition | 117.0007 | 2.33E-24 |
| R-HSA-176974 | Unwinding of DNA | 135.4972 | 2.60E-28 |
| R-HSA-195258 | RHO GTPase Effectors | 141.5633 | 1.31E-29 |
| R-HSA-69205 | G1/S-Specific Transcription | 113.2966 | 1.44E-23 |
| R-HSA-73894 | DNA Repair | 103.8048 | 1.52E-21 |
| R-HSA-5693538 | Homology Directed Repair | 113.476 | 1.32E-23 |
| R-HSA-453274 | Mitotic G2-G2/M phases | 92.73333 | 3.46E-19 |
| R-HSA-5693532 | DNA Double-Strand Break Repair | 104.4173 | 1.13E-21 |
| R-HSA-5693567 | HDR through Homologous Recombination (HRR) or Single Strand Annealing (SSA) | 102.225 | 3.30E-21 |
| R-HSA-69275 | G2/M Transition | 80.86156 | 1.14E-16 |
| R-HSA-156711 | Polo-like kinase mediated events | 86.58451 | 6.99E-18 |
| R-HSA-1538133 | G0 and Early G1 | 84.34096 | 2.09E-17 |
| R-HSA-5685942 | HDR through Homologous Recombination (HRR) | 86.02976 | 9.17E-18 |
| R-HSA-606279 | Deposition of new CENPA-containing nucleosomes at the centromere | 91.98024 | 5.00E-19 |
| R-HSA-774815 | Nucleosome assembly | 91.98024 | 5.00E-19 |
| R-HSA-69002 | DNA Replication Pre-Initiation | 112.4093 | 2.23E-23 |
| R-HSA-2514853 | Condensation of Prometaphase Chromosomes | 68.18535 | 5.48E-14 |
| R-HSA-9709603 | Impaired BRCA2 binding to PALB2 | 61.66042 | 1.30E-12 |
| R-HSA-9701192 | Defective homologous recombination repair (HRR) due to BRCA1 loss of function | 60.19789 | 2.64E-12 |
| R-HSA-9701193 | Defective homologous recombination repair (HRR) due to PALB2 loss of function | 60.19789 | 2.64E-12 |
| R-HSA-9704331 | Defective HDR through Homologous Recombination Repair (HRR) due to PALB2 loss of BRCA1 binding function | 60.19789 | 2.64E-12 |
| R-HSA-9704646 | Defective HDR through Homologous Recombination Repair (HRR) due to PALB2 loss of BRCA2/RAD51/RAD51C binding function | 60.19789 | 2.64E-12 |
| R-HSA-983189 | Kinesins | 74.88479 | 2.11E-15 |
| R-HSA-9675136 | Diseases of DNA Double-Strand Break Repair | 67.76018 | 6.74E-14 |
| R-HSA-9701190 | Defective homologous recombination repair (HRR) due to BRCA2 loss of function | 67.76018 | 6.74E-14 |
| R-HSA-9675135 | Diseases of DNA repair | 65.82465 | 1.72E-13 |
| R-HSA-5693554 | Resolution of D-loop Structures through Synthesis-Dependent Strand Annealing (SDSA) | 57.30619 | 1.07E-11 |
| R-HSA-5693579 | Homologous DNA Pairing and Strand Exchange | 69.77383 | 2.53E-14 |
| R-HSA-5693568 | Resolution of D-loop Structures through Holliday Junction Intermediates | 55.51105 | 2.54E-11 |
| R-HSA-5693537 | Resolution of D-Loop Structures | 54.42963 | 4.28E-11 |
| R-HSA-6791312 | TP53 Regulates Transcription of Cell Cycle Genes | 52.27287 | 1.21E-10 |
| R-HSA-5693616 | Presynaptic phase of homologous DNA pairing and strand exchange | 63.99788 | 4.18E-13 |
| R-HSA-180786 | Extension of Telomeres | 65.38834 | 2.13E-13 |
| R-HSA-174143 | APC/C-mediated degradation of cell cycle proteins | 54.76135 | 3.65E-11 |
| R-HSA-453276 | Regulation of mitotic cell cycle | 54.76135 | 3.65E-11 |
| R-HSA-174417 | Telomere C-strand (Lagging Strand) Synthesis | 66.8003 | 1.07E-13 |
| R-HSA-9709570 | Impaired BRCA2 binding to RAD51 | 55.79645 | 2.21E-11 |
| R-HSA-69052 | Switching of origins to a post-replicative state | 59.21187 | 4.25E-12 |
| R-HSA-5651801 | PCNA-Dependent Long Patch Base Excision Repair | 60.9524 | 1.83E-12 |
| R-HSA-68949 | Orc1 removal from chromatin | 51.36933 | 1.87E-10 |
| R-HSA-69473 | G2/M DNA damage checkpoint | 58.20793 | 6.90E-12 |
| R-HSA-5693607 | Processing of DNA double-strand break ends | 60.94325 | 1.84E-12 |
| R-HSA-9659787 | Aberrant regulation of mitotic G1/S transition in cancer due to RB1 defects | 46.17926 | 2.26E-09 |
| R-HSA-9661069 | Defective binding of RB1 mutants to E2F1,(E2F2, E2F3) | 46.17926 | 2.26E-09 |
| R-HSA-110373 | Resolution of AP sites via the multiple-nucleotide patch replacement pathway | 52.69737 | 9.86E-11 |
| R-HSA-174411 | Polymerase switching on the C-strand of the telomere | 47.93186 | 9.75E-10 |
| R-HSA-1362277 | Transcription of E2F targets under negative control by DREAM complex | 42.81459 | 1.13E-08 |
| R-HSA-69231 | Cyclin D associated events in G1 | 40.68427 | 3.12E-08 |
| R-HSA-69236 | G1 Phase | 40.68427 | 3.12E-08 |
| R-HSA-5685938 | HDR through Single Strand Annealing (SSA) | 44.90482 | 4.16E-09 |
| R-HSA-69186 | Lagging Strand Synthesis | 57.32618 | 1.06E-11 |
| R-HSA-157579 | Telomere Maintenance | 55.96782 | 2.04E-11 |
| R-HSA-6811434 | COPI-dependent Golgi-to-ER retrograde traffic | 46.75477 | 1.72E-09 |
| R-HSA-69091 | Polymerase switching | 46.0529 | 2.40E-09 |
| R-HSA-69109 | Leading Strand Synthesis | 46.0529 | 2.40E-09 |
| R-HSA-912446 | Meiotic recombination | 64.4815 | 3.31E-13 |
| R-HSA-110314 | Recognition of DNA damage by PCNA-containing replication complex | 47.6384 | 1.12E-09 |
| R-HSA-113510 | E2F mediated regulation of DNA replication | 43.51214 | 8.10E-09 |
| R-HSA-1500620 | Meiosis | 58.8849 | 4.97E-12 |
| R-HSA-6804756 | Regulation of TP53 Activity through Phosphorylation | 47.03393 | 1.50E-09 |
| R-HSA-1362300 | Transcription of E2F targets under negative control by p107 (RBL1) and p130 (RBL2) in complex with HDAC1 | 37.02109 | 1.78E-07 |
| R-HSA-9687139 | Aberrant regulation of mitotic cell cycle due to RB1 defects | 38.28804 | 9.77E-08 |
| R-HSA-6804114 | TP53 Regulates Transcription of Genes Involved in G2 Cell Cycle Arrest | 34.01224 | 7.41E-07 |
| R-HSA-73893 | DNA Damage Bypass | 39.63125 | 5.16E-08 |
| R-HSA-9675126 | Diseases of mitotic cell cycle | 36.57735 | 2.20E-07 |
| R-HSA-2995383 | Initiation of Nuclear Envelope (NE) Reformation | 37.5402 | 1.39E-07 |
| R-HSA-176814 | Activation of APC/C and APC/C:Cdc20 mediated degradation of mitotic proteins | 33.71796 | 8.51E-07 |
| R-HSA-73933 | Resolution of Abasic Sites (AP sites) | 38.84577 | 7.50E-08 |
| R-HSA-73884 | Base Excision Repair | 44.36889 | 5.38E-09 |
| R-HSA-1474165 | Reproduction | 51.02251 | 2.21E-10 |
| R-HSA-2559583 | Cellular Senescence | 52.61719 | 1.02E-10 |
| R-HSA-176408 | Regulation of APC/C activators between G1/S and early anaphase | 31.61412 | 2.29E-06 |
| R-HSA-3700989 | Transcriptional Regulation by TP53 | 27.61751 | 1.49E-05 |
| R-HSA-5656169 | Termination of translesion DNA synthesis | 36.81071 | 1.97E-07 |
| R-HSA-6804116 | TP53 Regulates Transcription of Genes Involved in G1 Cell Cycle Arrest | 29.58255 | 5.95E-06 |
| R-HSA-69166 | Removal of the Flap Intermediate | 34.71138 | 5.32E-07 |
| R-HSA-68867 | Assembly of the pre-replicative complex | 56.73254 | 1.41E-11 |
| R-HSA-8856688 | Golgi-to-ER retrograde transport | 35.36142 | 3.92E-07 |
| R-HSA-983231 | Factors involved in megakaryocyte development and platelet production | 49.37497 | 4.88E-10 |
| R-HSA-69183 | Processive synthesis on the lagging strand | 38.55494 | 8.61E-08 |
| R-HSA-5696397 | Gap-filling DNA repair synthesis and ligation in GG-NER | 39.70531 | 4.98E-08 |
| R-HSA-176409 | APC/C:Cdc20 mediated degradation of mitotic proteins | 27.9296 | 1.29E-05 |
| R-HSA-5633007 | Regulation of TP53 Activity | 29.28793 | 6.83E-06 |
| R-HSA-110313 | Translesion synthesis by Y family DNA polymerases bypasses lesions on DNA template | 31.07013 | 2.96E-06 |
| R-HSA-9825892 | Regulation of MITF-M-dependent genes involved in cell cycle and proliferation | 20.86567 | 3.37E-04 |
| R-HSA-6783310 | Fanconi Anemia Pathway | 26.83511 | 2.15E-05 |
| R-HSA-2565942 | Regulation of PLK1 Activity at G2/M Transition | 22.85383 | 1.35E-04 |
| R-HSA-2299718 | Condensation of Prophase Chromosomes | 41.73722 | 1.89E-08 |
| R-HSA-8854518 | AURKA Activation by TPX2 | 23.06206 | 1.23E-04 |
| R-HSA-2559580 | Oxidative Stress Induced Senescence | 38.80581 | 7.64E-08 |
| R-HSA-2559585 | Oncogene Induced Senescence | 27.85077 | 1.34E-05 |
| R-HSA-69656 | Cyclin A:Cdk2-associated events at S phase entry | 17.8984 | 0.001292 |
| R-HSA-8953750 | Transcriptional Regulation by E2F6 | 22.89675 | 1.33E-04 |
| R-HSA-162658 | Golgi Cisternae Pericentriolar Stack Reorganization | 19.85334 | 5.34E-04 |
| R-HSA-179409 | APC-Cdc20 mediated degradation of Nek2A | 22.37634 | 1.69E-04 |
| R-HSA-75035 | Chk1/Chk2(Cds1) mediated inactivation of Cyclin B:Cdk1 complex | 23.19349 | 1.16E-04 |
| R-HSA-68875 | Mitotic Prophase | 32.98364 | 1.20E-06 |
| R-HSA-9645723 | Diseases of programmed cell death | 36.00922 | 2.88E-07 |
| R-HSA-2132295 | MHC class II antigen presentation | 19.20703 | 7.16E-04 |
| R-HSA-5696400 | Dual Incision in GG-NER | 22.72259 | 1.44E-04 |
| R-HSA-69202 | Cyclin E associated events during G1/S transition | 15.67422 | 0.003489 |
| R-HSA-179419 | APC:Cdc20 mediated degradation of cell cycle proteins prior to satisfation of the cell cycle checkpoint | 17.1182 | 0.001833 |
| R-HSA-4615885 | SUMOylation of DNA replication proteins | 17.58219 | 0.001489 |
| R-HSA-174414 | Processive synthesis on the C-strand of the telomere | 23.52236 | 9.96E-05 |
| R-HSA-176412 | Phosphorylation of the APC/C | 19.29013 | 6.89E-04 |
| R-HSA-2559586 | DNA Damage/Telomere Stress Induced Senescence | 23.36221 | 1.07E-04 |
| R-HSA-141405 | Inhibition of the proteolytic activity of APC/C required for the onset of anaphase by mitotic spindle checkpoint components | 18.51335 | 9.79E-04 |
| R-HSA-141430 | Inactivation of APC/C via direct inhibition of the APC/C complex | 18.51335 | 9.79E-04 |
| R-HSA-6811442 | Intra-Golgi and retrograde Golgi-to-ER traffic | 17.72813 | 0.001395 |
| R-HSA-9856651 | MITF-M-dependent gene expression | 15.95756 | 0.003077 |
| R-HSA-2980766 | Nuclear Envelope Breakdown | 14.82109 | 0.005087 |
| R-HSA-174048 | APC/C:Cdc20 mediated degradation of Cyclin B | 16.28622 | 0.002658 |
| R-HSA-5685939 | HDR through MMEJ (alt-NHEJ) | 13.18562 | 0.010404 |
| R-HSA-9013405 | RHOD GTPase cycle | 14.10608 | 0.006964 |
| R-HSA-9710421 | Defective pyroptosis | 33.25124 | 1.06E-06 |
| R-HSA-2559582 | Senescence-Associated Secretory Phenotype (SASP) | 31.57711 | 2.33E-06 |
| R-HSA-9013106 | RHOC GTPase cycle | 17.38842 | 0.001624 |
| R-HSA-9013148 | CDC42 GTPase cycle | 12.9846 | 0.011351 |
| R-HSA-2995410 | Nuclear Envelope (NE) Reassembly | 14.70049 | 0.005364 |
| R-HSA-5358565 | Mismatch repair (MMR) directed by MSH2:MSH6 (MutSalpha) | 19.93161 | 5.15E-04 |
| R-HSA-5358606 | Mismatch repair (MMR) directed by MSH2:MSH3 (MutSbeta) | 19.93161 | 5.15E-04 |
| R-HSA-5693565 | Recruitment and ATM-mediated phosphorylation of repair and signaling proteins at DNA double strand breaks | 12.1996 | 0.015927 |
| R-HSA-174184 | Cdc20:Phospho-APC/C mediated degradation of Cyclin A | 12.66395 | 0.01304 |

**Table 8. Summary of genes identified through network topology analysis as commonly upregulated or downregulated across acute and replicative senescence conditions. These nodes represent key hubs and connectors within the shared senescence-associated interaction network.**

| **Common Upregulated network** | **Common Downregulated network** |
| --- | --- |
| UBC | KIAA0101 |
| CCND1 | UBC |
| APP | CDK2 |
| SPP1 | BRCA1 |
| ELF3 | BARD1 |
| APLP1 | PLK1 |
| HNF4A | HIST1H3H |
| ATP13A2 | TK1 |
| ELAVL1 | EZH2 |
| DYSF | CDK1 |
| TNFRSF10D | APP |
| DYNC1I1 | SUMO2 |
| INPP5D | MCM7 |
| LRP2 | PTN |
| MAST1 | HNF4A |
| CCL5 | MCM3 |
| GRB2 | CCNB1 |
| CSF2 | RBL1 |
| CHGB | MYB |
| CCL2 | CDC20 |
| SP1 | HIST2H3C |
| ABCB1 | MCM2 |
| EGR1 | HIST2H2AC |
| C3 | CUL3 |
| HSP90AA1 | AURKA |
| PKP2 | CUL1 |
| CCND2 | FN1 |
| FOXA1 | CCNA2 |
| TP53 | COPS5 |
| CDKN1A | ESR1 |
| RB1 | AURKB |
| GADD45A | SUMO1 |
| TNF | RAD51 |
| HMGA1 | CENPA |
| IL6 | ELAVL1 |
| CASP8 | TRIP13 |
| PLAUR | ITGA4 |
| ATM | MYC |
| MMP9 | UBE2C |
| FAS | NEDD8 |
| CDKN2A | SOCS1 |
| VCAM1 | YWHAZ |
| BAX | BRCA2 |
| IL1B | TOP2A |
| FASLG | TP53 |
|  | POU5F1 |
|  | CDC25A |
|  | LMNB1 |
|  | RFC4 |
|  | SIRT7 |
|  | BLM |
|  | MCM5 |
|  | BUB1B |
|  | HIST1H1A |
|  | TCF4 |
|  | CDT1 |
|  | HDAC1 |
|  | TMPO |
|  | EGR1 |
|  | SP1 |
|  | CDC6 |
|  | HSP90AA1 |
|  | MAD2L1 |
|  | FKBP5 |
|  | DEK |
|  | ITGB3BP |
|  | HSP90AB1 |
|  | BIRC5 |
|  | RUNX1T1 |
|  | H2AFZ |
|  | ACTC1 |
|  | TRAIP |
|  | FANCD2 |
|  | SMC2 |
|  | TYMS |
|  | RPL10L |
|  | PBX1 |
|  | AMPH |
|  | EP300 |
|  | FANCG |
|  | ZWINT |
|  | PSIP1 |
|  | HIST1H1B |
|  | CBX2 |
|  | FEN1 |
|  | USP1 |
|  | MCM10 |
|  | ORC1 |
|  | FANCI |
|  | PTTG1 |
|  | MYBL2 |
|  | ASF1B |
|  | YWHAQ |
|  | POLA1 |
|  | NFIA |
|  | VCP |
|  | GRB2 |
|  | BUB1 |
|  | HIST1H2BE |
|  | PCNA |
|  | SHMT1 |
|  | NEK2 |
|  | SIRT1 |
|  | CDC45 |
|  | FOXM1 |
|  | TPX2 |
|  | SMC4 |
|  | DTL |
|  | DES |
|  | STMN1 |
|  | CDC25C |
|  | CTNNB1 |
|  | MLF1IP |
|  | NDC80 |
|  | HDAC2 |
|  | HMGB2 |
|  | KIF23 |
|  | CDC7 |
|  | CDKN2C |
|  | H2AFX |
|  | HIST1H2BB |
|  | FBXO5 |
|  | RACGAP1 |
|  | GINS3 |
|  | CDKN1A |
|  | CKS1B |
|  | GINS4 |
|  | RFC3 |
|  | SNCAIP |
|  | MKI67 |
|  | RPA1 |
|  | GMNN |
|  | EXOSC9 |
|  | FZR1 |
|  | NCAPG |
|  | CLSPN |
|  | HIST1H1E |
|  | EXO1 |
|  | MAP2K6 |
|  | TACC3 |
|  | XRCC6 |
|  | E2F1 |
|  | ORC6 |
|  | PBK |
|  | CENPF |
|  | E2F2 |
|  | PITX2 |
|  | HIST1H1D |
|  | PARP1 |

**Table 9. Computational Environment (Session Info) Used to Ensure Reproducibility of the Analysis**

| **Category** | **Library** | **Version** | **Purpose** |
| --- | --- | --- | --- |
| **Core Normalization & Batch Correction** | DESeq2 | 1.48.1 | Variance-stabilized transformation and normalization |
|  | limma | 3.64.3 | Batch-effect removal and linear modeling |
|  | sva | 3.56.0 | Surrogate variable estimation for hidden confounders |
| **Data Handling & Transformation** | tidyverse | 2.0.0 | Unified data manipulation suite |
|  | dplyr | 1.1.4 | Data wrangling and filtering |
|  | readr | 2.1.5 | CSV input/output |
|  | tibble | 3.3.0 | Modern data frames |
|  | purrr | 1.1.0 | Functional iteration |
|  | stringr | 1.5.2 | String processing |
| **Visualization** | ggplot2 | 4.0.0 | Plot generation (PCA, volcano, bar plots) |
|  | ggrepel | 0.9.6 | Non-overlapping text labels |
|  | pheatmap | 1.0.13 | Expression heatmaps |
|  | VennDiagram | 1.7.3 | Classical Venn diagrams |
|  | ggvenn | 0.1.10 | ggplot-based Venn visualization |
|  | grid | Base | Low-level graphics support |
| **Functional Enrichment** | clusterProfiler | 4.16.0 | GO Biological Process enrichment |
|  | ReactomePA | 1.52.0 | Reactome pathway analysis |
|  | org.Hs.eg.db | 3.21.0 | Human gene annotation |
| **Bioconductor Infrastructure** | Biobase | 2.68.0 | Core data structures |
|  | SummarizedExperiment | 1.38.1 | Expression container |
|  | MatrixGenerics | 1.20.0 | Matrix operations |
|  | IRanges | 2.42.0 | Genomic range manipulation |
|  | S4Vectors | 0.46.0 | Object support for S4 classes |
|  | GenomeInfoDb | 1.44.2 | Genomic metadata management |

**Table 10. Global batch-correction metrics (before vs after SVA)**

| **Stage** | **Mean silhouette (ALL)** | **Mean within distance** | **Mean between distance** | **Within / Between** |
| --- | --- | --- | --- | --- |
| Before | 0.0636 | 144.33 | 173.93 | 0.8299 |
| After | 0.3787 | 43.64 | 77.21 | 0.5653 |

**Table 11. Condition-specific silhouette scores**

| **Stage** | **Control** | **Replicative senescence** | **Acute senescence** |
| --- | --- | --- | --- |
| Before | 0.028 | 0.164 | 0.003 |
| After | 0.480 | 0.310 | 0.270 |

**Table 12. kNN batch-mixing scores (fraction of same-batch neighbors)**

| **Stage** | **Control** | **Replicative senescence** | **Acute senescence** |
| --- | --- | --- | --- |
| Before | 0.60 | 0.36 | 0.68 |
| After | 0.27 | 0.27 | 0.36 |
